# Supplementary material for: Interaction between transcribing RNA polymerase and topoisomerase I prevents R-loop formation in E. coli
Source: Nat Commun. 2022 Aug 4;13:4524. doi: 10.1038/s41467-022-32106-5 (PMC9352719; doi:10.1038/s41467-022-32106-5)
Supplement: Supplementary file 1 — Supplementary Information [file 41467_2022_32106_MOESM1_ESM.pdf]

Supplementary Information

Interaction between transcribing RNA polymerase and topoisomerase I prevents R-loop formation in *E. coli*

Dmitry Sutormin[1,2#], Alina Galivondzhyan[1,3], Olga Musharova[1,4], Dmitrii Travin[1,2], Anastasiya Rusanova[2], Kseniya Obraztsova[5,6], Sergei Borukhov[6], Konstantin Severinov[1,7,8#]

[1]Skolkovo Institute of Science and Technology, Moscow, 121205 Russia

[2]Institute of Gene Biology RAS, Moscow, 119334 Russia

[3]Lomonosov Moscow States University, Moscow, 119991 Russia

[4]Institute of Molecular Genetics, National Research Centre "Kurchatov Institute," Moscow 123182, Russia

[5]Current address: University of Pennsylvania, Perelman School of Medicine, Department of Medicine, Philadelphia, PA 19104, United States

[6]Department of Cell Biology and Neuroscience, Rowan University School of Osteopathic Medicine, Stratford, NJ 08084-1489, USA

[7]Center for Precision Genome Editing and Genetic Technologies for Biomedicine, Institute of Gene Biology, RAS, Moscow, 119334 Russia

[8]Waksman University for Microbiology, Rutgers, NJ 08854, USA

#To whom correspondence should be addressed. E-mail: K.Severinov@skoltech.ru, D.A.Sutormin@gmail.com

Supplementary Figures

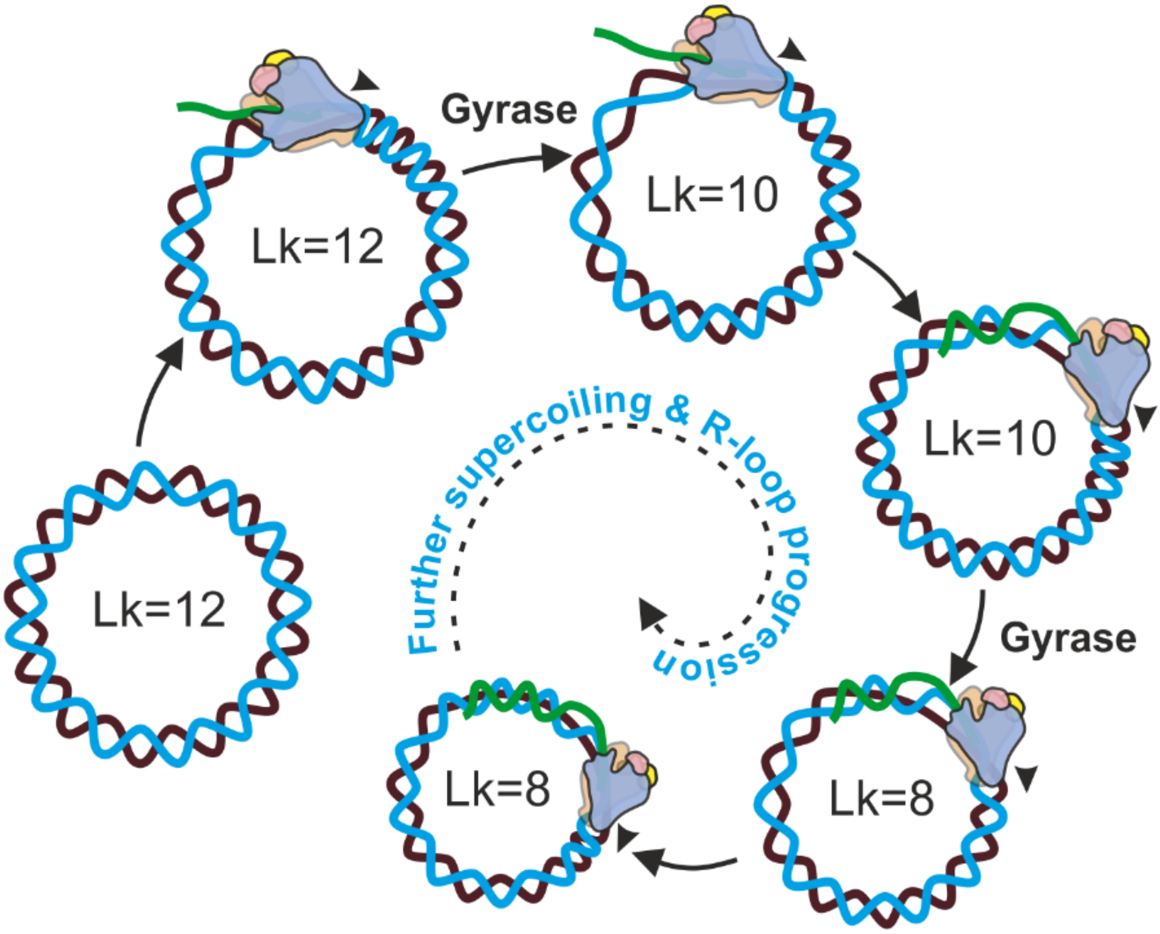

Supplementary Figure 1. A positive feedback cycle leads to hypernegative supercoiling of plasmid DNA and R-loops accumulation when transcription proceeds in the presence of DNA gyrase and the absence of TopoI. RNAP is shown as a cartoonish protein complex. Direction of RNAP elongation is shown with an arrow. Nascent RNA is shown in green. Overwinding of RNA with a blue DNA strand (template strand) indicates formation of an R-loop. Decreased and increased density of DNA turns behind and ahead of elongating RNAP corresponds to negative and positive supercoiling, respectively. Gyrase indicates negative supercoiling by gyrase. Lk, linking number. Decreasing linking number corresponds to increasing level of negative supercoiling of a plasmid over time.

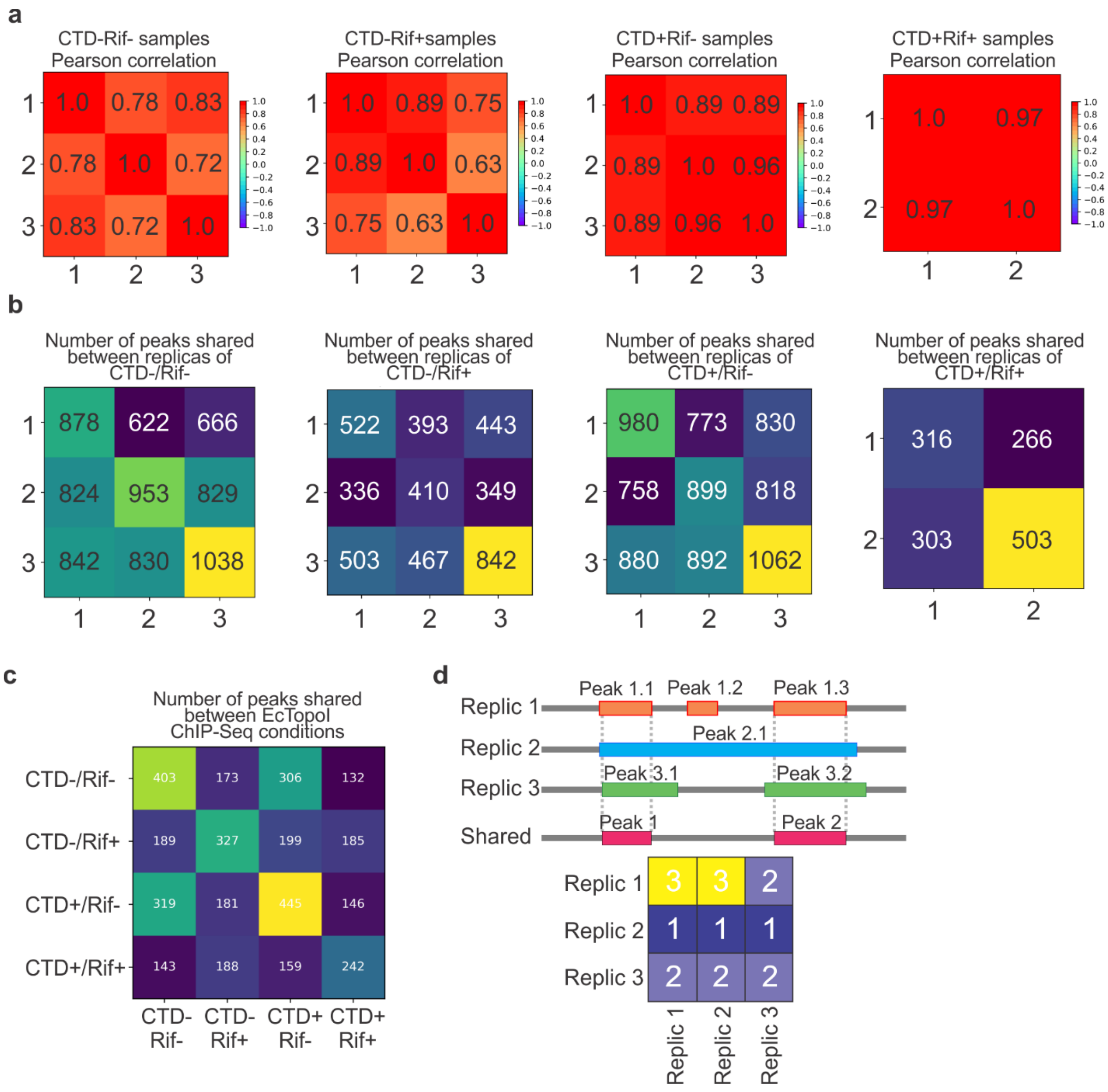
a
CTD-Rif- samples
Pearson correlation
1.0 0.78 0.83
0.78 1.0 0.72
0.83 0.72 1.0
CTD-Rif+samples
Pearson correlation
1.0 0.89 0.75
0.89 1.0 0.63
0.75 0.63 1.0
CTD+Rif- samples
Pearson correlation
1.0 0.89 0.89
0.89 1.0 0.96
0.89 0.96 1.0
CTD+Rif+ samples
Pearson correlation
1.0 0.97
0.97 1.0
b
Number of peaks shared between replicas of CTD-/Rif-
878 622 666
824 953 829
842 830 1038
Number of peaks shared between replicas of CTD-/Rif+
522 393 443
336 410 349
503 467 842
Number of peaks shared between replicas of CTD+/Rif-
980 773 830
758 899 818
880 892 1062
Number of peaks shared between replicas of CTD+/Rif+
316 266
303 503
c
Number of peaks shared between EcTopoI ChIP-Seq conditions
CTD-/Rif- 403 173 306 132
CTD-/Rif+ 189 327 199 185
CTD+/Rif- 319 181 445 146
CTD+/Rif+ 143 188 159 242
CTD- Rif-
CTD- Rif+
CTD+ Rif-
CTD+ Rif+
d
Replic 1
Replic 2
Replic 3
Shared
Peak 1.1
Peak 1.2
Peak 1.3
Peak 2.1
Peak 3.1
Peak 3.2
Peak 1
Peak 2
Replic 1 3 3 2
Replic 2 1 1 1
Replic 3 2 2 2

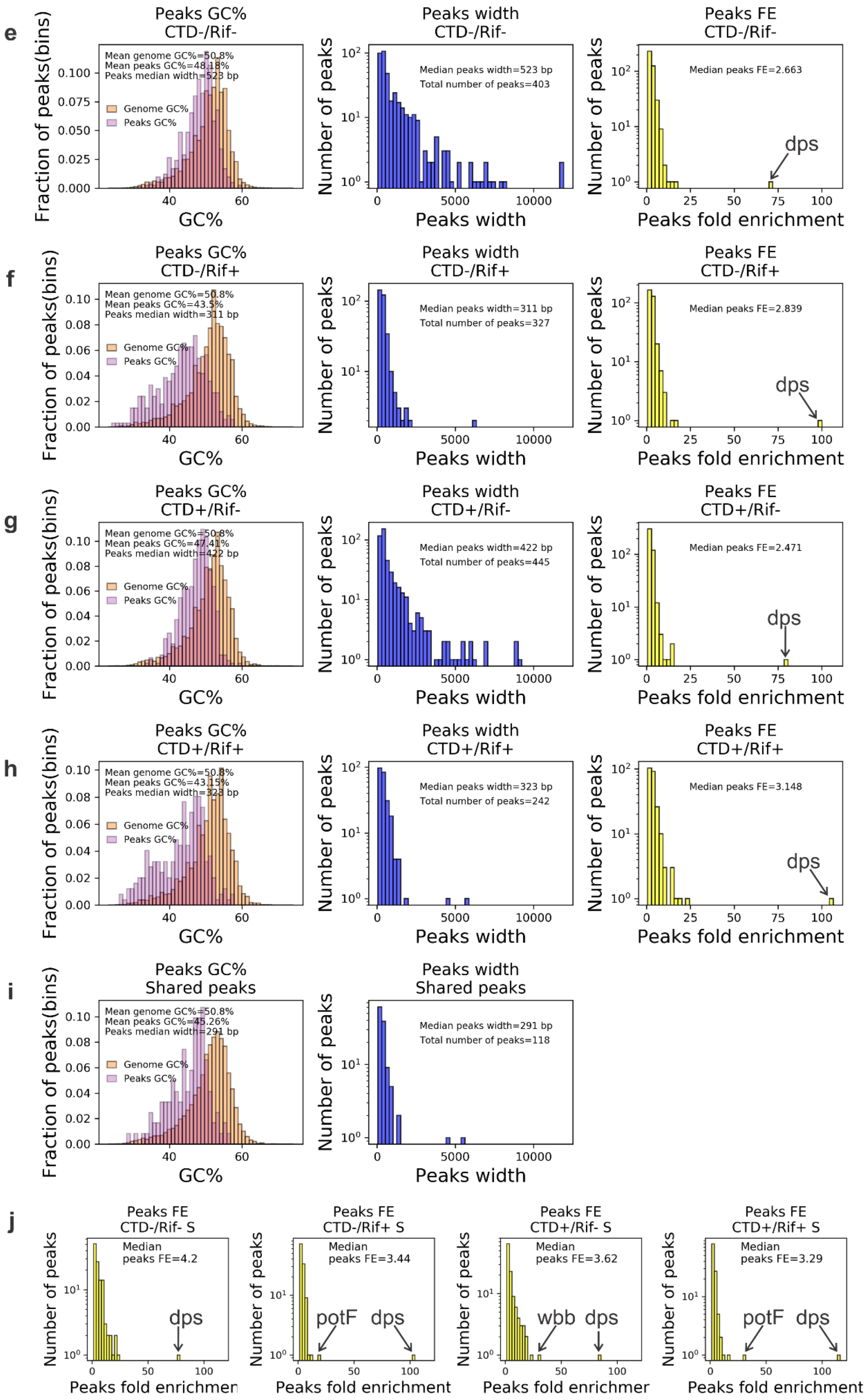
e
Peaks GC%
CTD-/Rif-
Mean genome GC%=50.8%
Mean peaks GC%=48.18%
Peaks median width=523 bp
Genome GC%
Peaks GC%
Fraction of peaks(bins)
GC%
Peaks width
CTD-/Rif-
Median peaks width=523 bp
Total number of peaks=403
Number of peaks
Peaks width
Peaks FE
CTD-/Rif-
Median peaks FE=2.663
dps
Peaks fold enrichment
f
Peaks GC%
CTD-/Rif+
Mean genome GC%=50.8%
Mean peaks GC%=43.5%
Peaks median width=311 bp
Peaks width
CTD-/Rif+
Median peaks width=311 bp
Total number of peaks=327
Peaks FE
CTD-/Rif+
Median peaks FE=2.839
dps
g
Peaks GC%
CTD+/Rif-
Mean genome GC%=50.8%
Mean peaks GC%=47.41%
Peaks median width=422 bp
Peaks width
CTD+/Rif-
Median peaks width=422 bp
Total number of peaks=445
Peaks FE
CTD+/Rif-
Median peaks FE=2.471
dps
h
Peaks GC%
CTD+/Rif+
Mean genome GC%=50.8%
Mean peaks GC%=43.15%
Peaks median width=323 bp
Peaks width
CTD+/Rif+
Median peaks width=323 bp
Total number of peaks=242
Peaks FE
CTD+/Rif+
Median peaks FE=3.148
dps
i
Peaks GC%
Shared peaks
Mean genome GC%=50.8%
Mean peaks GC%=45.26%
Peaks median width=291 bp
Peaks width
Shared peaks
Median peaks width=291 bp
Total number of peaks=118
j
Peaks FE
CTD-/Rif- S
Median
peaks FE=4.2
dps
Peaks FE
CTD-/Rif+ S
Median
peaks FE=3.44
potF
dps
Peaks FE
CTD+/Rif- S
Median
peaks FE=3.62
wbb
dps
Peaks FE
CTD+/Rif+ S
Median
peaks FE=3.29
potF
dps

Supplementary Figure 2 (related to Figures 1, 2). Correspondence between biological replicas of EcTopoI ChIP-Seq experiments.

(**a**) Heatmaps representing pair-wise Pearson correlation between EcTopoI ChIP-Seq datasets (fold enrichment). From left to right: correlations within sets of CTD-Rif- (no EcTopoI 14kDa CTD overexpression, no Rif treatment), CTD-Rif+ (no EcTopoI 14kDa CTD overexpression, with Rif treatment), CTD+Rif- (EcTopoI 14kDa CTD overexpression, no Rif treatment), and CTD+Rif+ (EcTopoI 14kDa CTD overexpression, with Rif treatment) samples.

(**b**) Heatmaps represent the pair-wise numbers of enrichment peaks identified with MACS2 that are shared between EcTopoI ChIP-Seq datasets**.** From left to right: number of peaks shared within sets of CTD-/Rif-, CTD-/Rif+, CTD+/Rif-, and CTD+/Rif+ biological replicas.

(**c**) Numbers of EcTopoI peaks shared between different experimental conditions. Peaks identified in all biological replicas of a particular experimental condition were considered.

(**d**) Presented matrices of shared EcTopoI peaks are not symmetric because several enriched regions (peaks) of sample 1 can overlap with one long peak of sample 1. In this example, the number of shared peaks will be higher for sample 1 than for sample 2.

(**e**) CTD-/Rif- peaks. (**f**) CTD-/Rif+ peaks. (**g**) CTD+/Rif- peaks. (**h**) CTD+/Rif+ peaks. (**i**) Peaks shared between all datasets. (**j**) Fold enrichment of peaks shared between all datasets from different datasets (S – for shared).

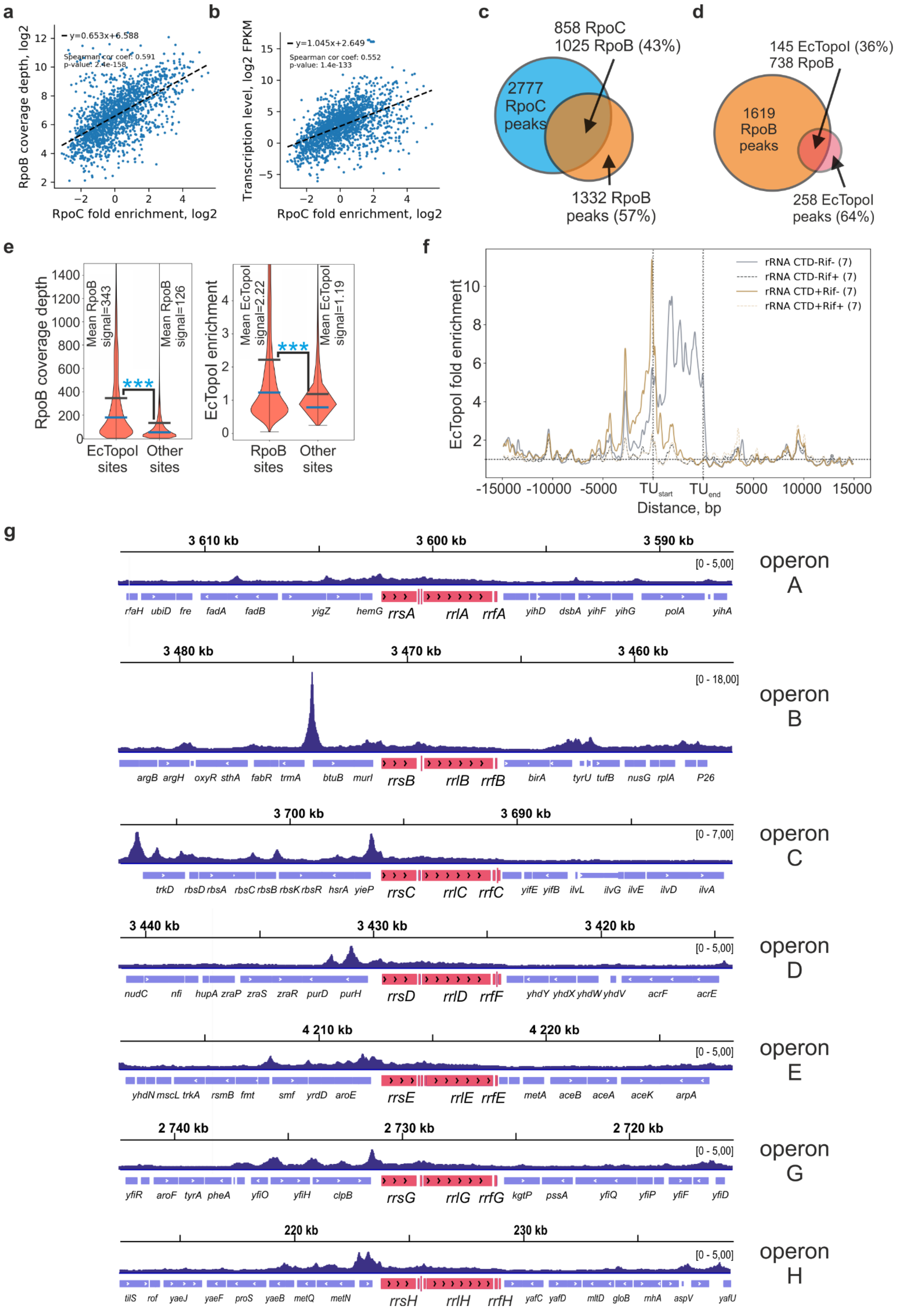

a
y=0.653x+6.588
Spearman cor coef: 0.591
p-value: 2.4e-158
RpoB coverage depth, log2
RpoC fold enrichment, log2
b
y=1.045x+2.649
Spearman cor coef: 0.552
p-value: 1.4e-133
Transcription level, log2 FPKM
RpoC fold enrichment, log2
c
858 RpoC
1025 RpoB (43%)
2777 RpoC peaks
1332 RpoB peaks (57%)
d
145 EcTopoI (36%)
738 RpoB
1619 RpoB peaks
258 EcTopoI peaks (64%)
e
RpoB coverage depth
Mean RpoB signal=343
Mean RpoB signal=126
EcTopoI sites
Other sites
EcTopoI enrichment
Mean EcTopoI signal=2.22
Mean EcTopoI signal=1.19
RpoB sites
Other sites
f
EcTopoI fold enrichment
rRNA CTD-Rif- (7)
rRNA CTD-Rif+ (7)
rRNA CTD+Rif- (7)
rRNA CTD+Rif+ (7)
Distance, bp
g
operon A
operon B
operon C
operon D
operon E
operon G
operon H

h

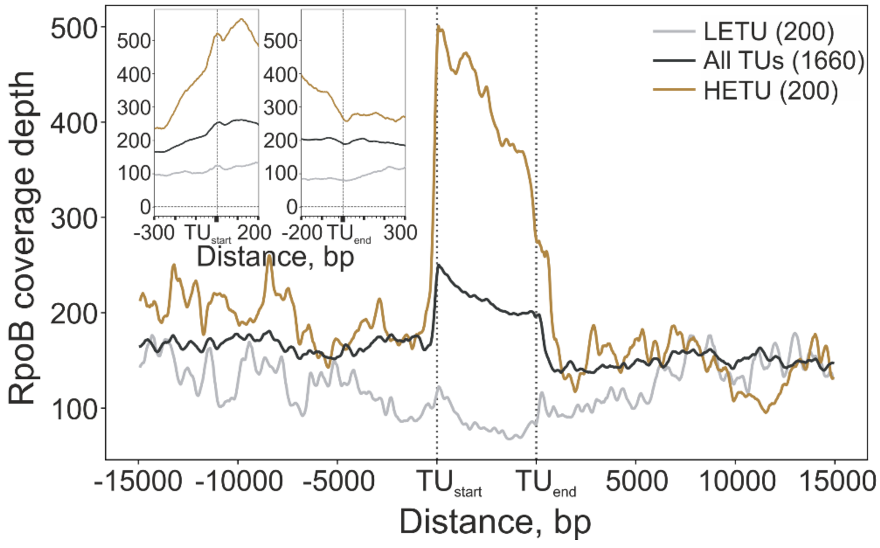

Supplementary Figure 3 (related to Figure 2). Associations between transcription, RNAP, TopoI, and DNA-gyrase in *E. coli*.

(**a**) Correlation between RpoC fold enrichment and RpoB signal for TUs (Spearman correlation coefficient, two-sided).

(**b**) Correlation between RpoC fold enrichment and transcription level of TUs (Spearman correlation coefficient, two-sided).

(**c**) Number of RpoC peaks (our data) overlapping with RpoB peaks (data from Kahramanoglou *et al.*, 2011 [1]). Venn diagram representing an overlap of RpoC peaks (3635 in total) and RpoB peaks (2357 in total). Raw sequencing data for RpoB (RNAP β-subunit) ChIP-Seq performed for exponentially growing *E. coli* MG1655 is taken from[1] and mapped on *E. coli* DY330 genome. Due to a lack of mock control for ChIP-Seq, regions with a coverage depth of > 450 were defined as RpoB peaks (coverage depth ~3x over background).

(**d**) Number of RpoB peaks overlapping with EcTopoI peaks. The Venn diagram represents an overlap of RpoB peaks (2357) and EcTopoI peaks (403).

(**e**) Violin plots of RpoB coverage depth in EcTopoI peak regions and other sites in *E. coli* genome (left), and EcTopoI enrichment in RpoB peaks regions and other sites in the genome (right). Mean and median are indicated by black and blue lines, respectively. The statistically significant differences between means (two-sided Welch t-test, p-value=5e-12 and 2e-42 for RpoB and EcTopoI enrichments, respectively) are indicated by asterisks.

(**f**) Metagene plot showing averaged fold enrichment over 7 rRNA operons. The solid gray line represents Rif-/CTD- ChIP-Seq experiments.

(**g**) Snapshots from IGV genomic browser representing EcTopoI fold enrichment around rRNA operons for Rif-/CTD- conditions. rRNA operons are listed from A to H; genes comprising the operons are colored in red. For panels F and G ChIP-Seq fold enrichment is given relative to the input sample.

(**h**) Signal of RpoB in TUs, their upstream (left) and downstream (right) regions. The metagene plot shows the distribution of the average ChIP-Seq signal for all TUs (All TUs), highly-expressed (HETU), and least-expressed (LETU) sets. The number of TUs used for analysis in each group is indicated in parentheses. The two insets show the zoom-in views of RpoB enrichment near transcription start and termination sites.

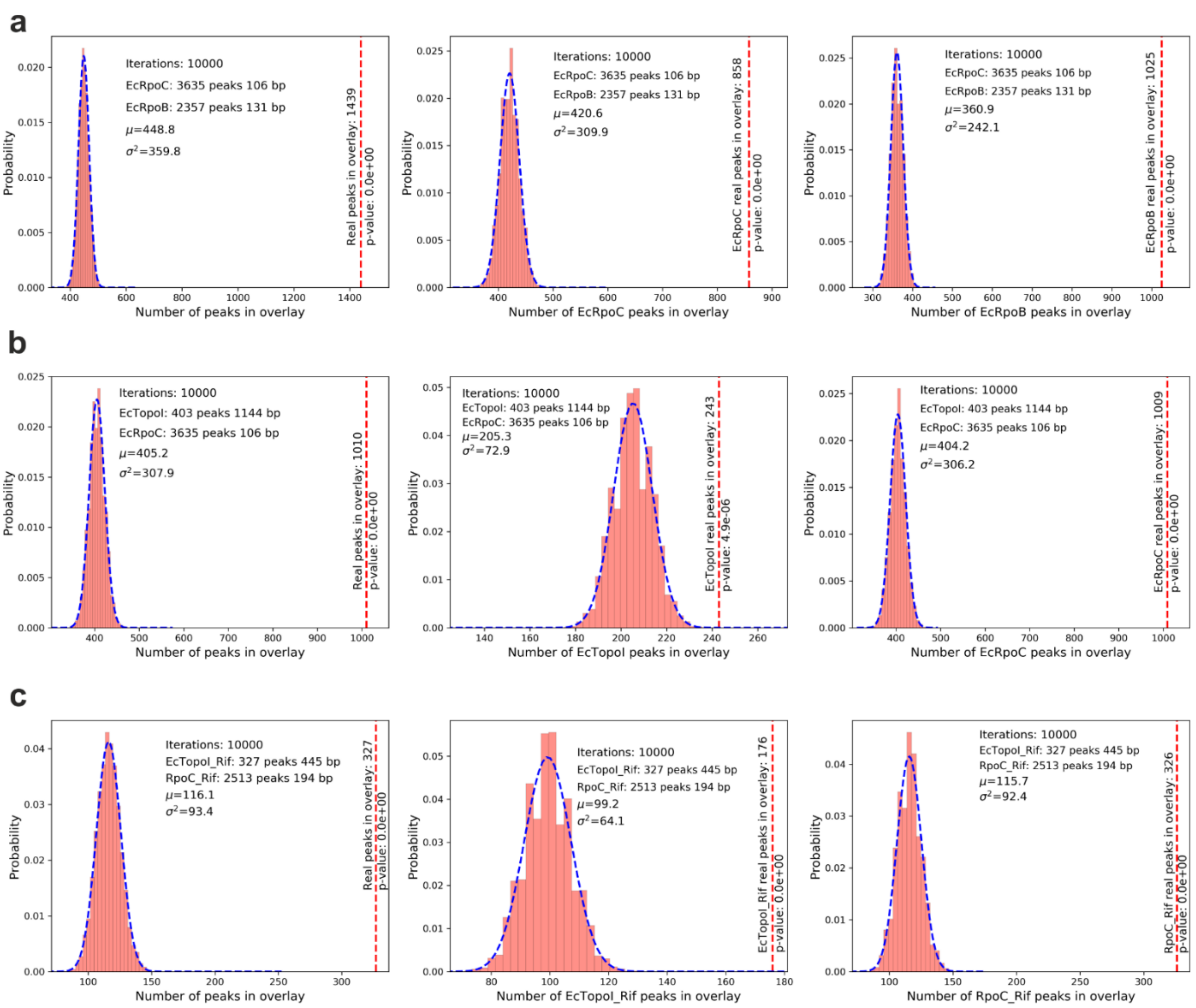

Supplementary Figure 4 (related to Figures 2). Monte-Carlo simulation of peak sets overlay. Sampling the overlay of:

(**a**) EcRpoC and EcRpoB peak sets,

(**b**) EcRNAP (RpoC) and EcTopoI peak sets,

(**c**) EcRNAP (RpoC) and EcTopoI peak sets for Rif+ condition.

Simulations were performed using the **Peak_overlap_simulation.py** script, 10000 simulations per test. For details, see ***Supplementary Methods***.

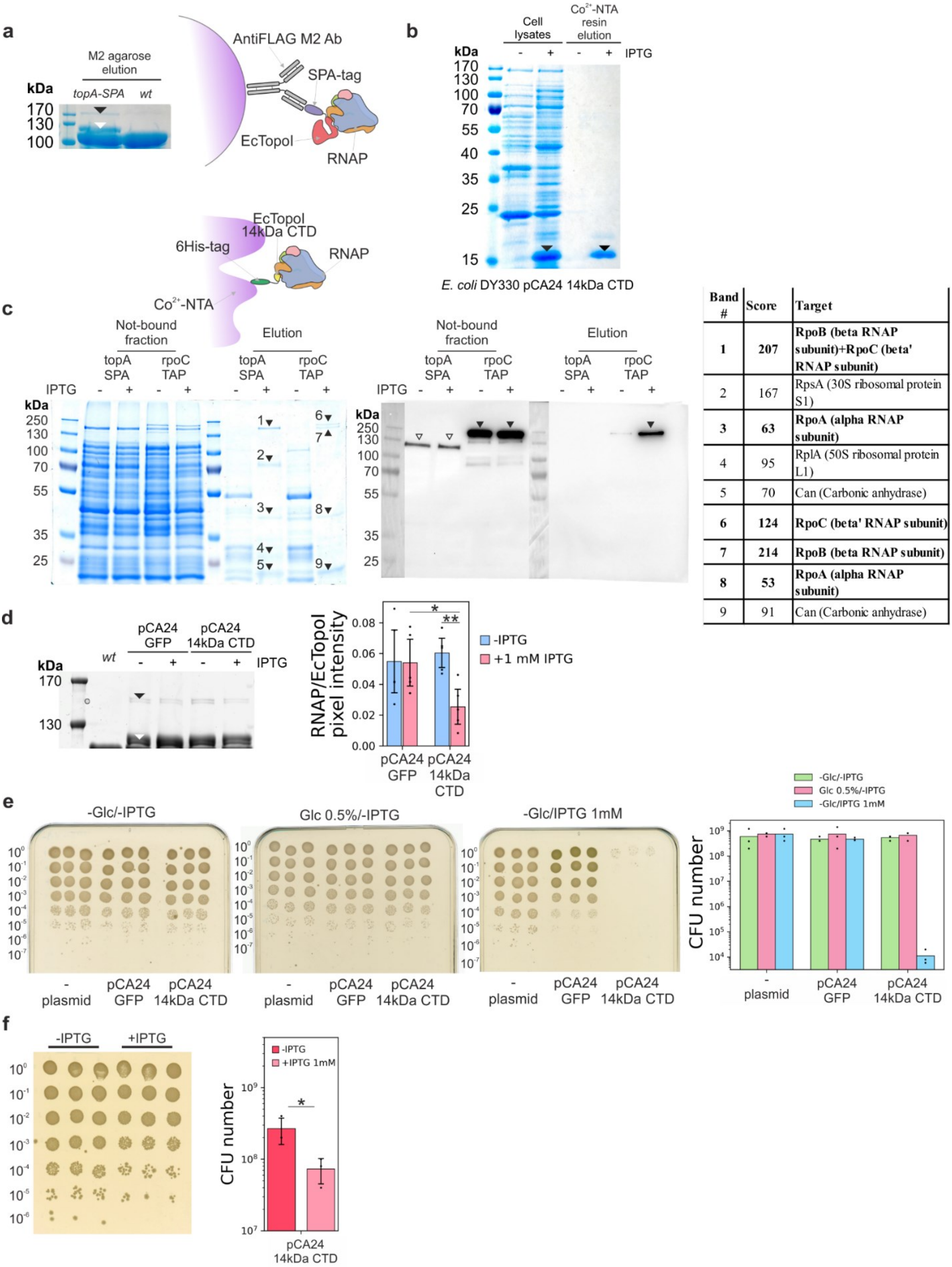

Band #	Score	Target
**1**	**207**	**RpoB (beta RNAP subunit)+RpoC (beta' RNAP subunit)**
2	167	RpsA (30S ribosomal protein S1)
**3**	**63**	**RpoA (alpha RNAP subunit)**
4	95	RplA (50S ribosomal protein L1)
5	70	Can (Carbonic anhydrase)
**6**	**124**	**RpoC (beta' RNAP subunit)**
**7**	**214**	**RpoB (beta RNAP subunit)**
**8**	**53**	**RpoA (alpha RNAP subunit)**
9	91	Can (Carbonic anhydrase)

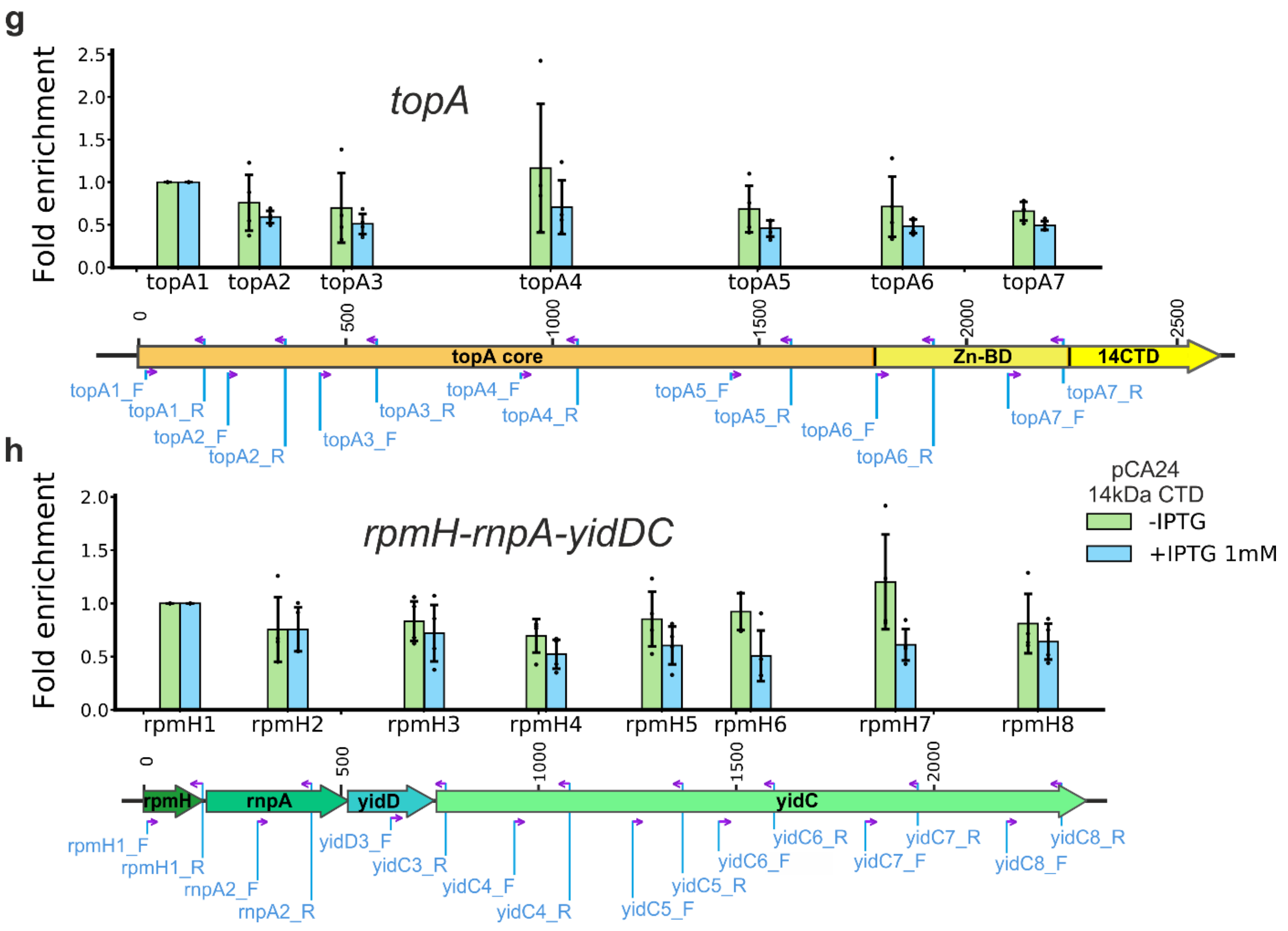

Supplementary Figure 5 (related to Figures 2 and 5).

(**a**) Pull-down experiment with *E. coli* DY330 *topA-SPA* using EcTopoI-SPA as a bait (see cartoon scheme on the right). Control experiment was performed with *E. coli* DY330 strain with non-tagged *topA* (*wt*). Pull-downs were analyzed by SDS-PAGE and protein bands were verified by MS – black triangle marks β and β' RNAP subunits, white triangle marks EcTopoI-SPA. Experiment was repeated three times and a representative gel is shown.

(**b**) Validation of EcTopoI 14kDa CTD protein overexpression from the pCA24 14kDa CTD plasmid by 12% SDS-PAGE. Coomassie-stained bands corresponding to 14kDa CTD (validated by MS) are marked with black triangles. Experiment was repeated three times and a representative gel is shown.

(**c**) Pull-down experiments with *E. coli* DY330 *topA-SPA* and *E. coli* DY330 *rpoC-TAP* strains using His-tagged CTD as bait (see cartoon scheme above). Protein bands were analyzed by MS (Coomassie-stained gel – on the left); RpoC-TAP and EcTopoI-SPA were detected by Western blotting (on the right) using primary mouse anti-FLAG MAbs and secondary rabbit anti-mouse Abs conjugated with HRP. Numbered black triangles indicate protein bands examined by MS on the left gel: RNAP β-subunit, bands 1 and 7; RNAP β'-subunit, bands 1 and 6; RNAP α-subunit, bands 3 and 8 (see table on the right). EcTopo-SPA and RpoC-TAP are marked on the right blot by white and black triangles, respectively. Experiment was repeated two times and representative gel and blot are shown.

(**d**) Pull-down experiments were performed with *E. coli* DY330 *topA-SPA* using EcTopoI-SPA as a bait as described in **a**, but GFP or 14kDa CTD were overexpressed from corresponding pCA24 plasmids. Pull-downs were analyzed by SDS-PAGE, stained with Coomassie and protein bands intensity was quantified using Photoshop. EcTopoI-SPA is marked with a white triangle; RNAP β and β' are marked with a black triangle. Resultant RNAP/EcTopoI ratios observed in 5 separate experiments are shown in the bar graph on the right. Bars and error bars represent mean ratios ±SEM. Differences between conditions were tested using the two-sided Welch t-test. P-values<0.05 are represented with asterisks with the number of asterisks indicating the significance level. For source data see **Table S2** in Source Data file.

(**e**) Long-term overexpression of 14kDa CTD is toxic for *E. coli*. CFU counting on LB-agar plates of *E. coli* DY330 transformed with pCA24 14kDa CTD, pCA24 GFP, or without plasmid (- plasmid) after overnight growth in LB (-Glc/-IPTG), and LB supplemented with either 0.5% Glc (Glc 0.5%/-IPTG) or 1mM IPTG for plasmid induction (-Glc/IPTG 1mM) (see ***Methods*** for details). The bar graph on the right represents the CFU numbers observed for different cultures. For source data see **Table S3** in Source Data file.

(**f**) Short-term overexpression of 14kDa CTD has a low toxic effect on *E. coli* growth. CFU counting on LB-agar plates of *E. coli* DY330 transformed with pCA24 14kDa CTD after 1h-induction with 1 mM IPTG, or without induction. The bar graph on the right shows the quantification of CFUs. Experiments in **e** and **f** were performed in triplicates. Error bars represent the 0.95 confidence interval for mean. Differences between conditions were tested using the two-sided Welch t-test (p-value=4.8e-2). For source data see **Table S4** in Source Data file.

(**g**) The toxicity of the 14 kDa CTD may be caused by transcription alterations or by mRNA degradation. We quantified RNAP enrichment by ChIP-qPCR over *topA* TU in *E. coli* DY330 cells harboring pCA24 CTD plasmid for uninduced condition and after the 1h induction of 14kDa CTD expression with 1mM IPTG (for primer sequences, see **Supplementary Table 1**). Bars and error bars represent mean fold enrichment (IP sample/mock sample) normalized to a 5'-proximal region of *topA* (topA1 pair of primers) ±SD. Bars colors correspond to experimental conditions: green – non-induced cells with pCA24 CTD repressed by 0.5% glucose, blue – cells harboring pCA24 CTD induced with 1mM IPTG. Bar pairs location correspond to the position of primer pairs in the transcription unit. Below is a map of the *topA* gene showing the 14 kDa- and 30 kDa CTD fragments and primer pairs indicated. Experiments were performed in 4 biological replicates. For source data, see **Tables S7** in Source Data file.

(**h**) The analysis as in **g**, but for *rpmH-rnpA-yidD-yidC* transcription unit. Experiments were performed in four biological replicates. For source data, see **Tables S7** in Source Data file.

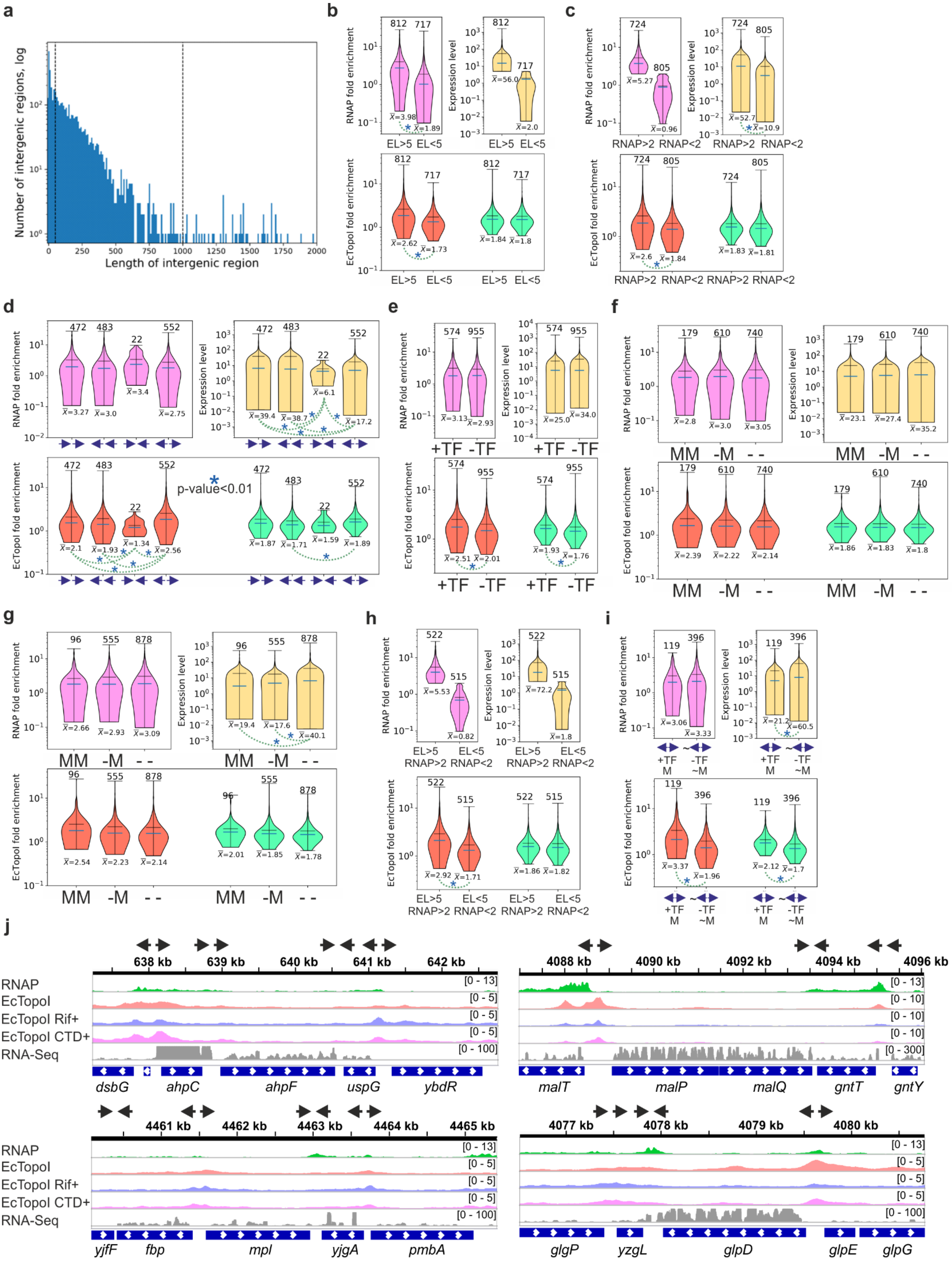
a
Number of intergenic regions, log
Length of intergenic region
b
RNAP fold enrichment
Expression level
EcTopoI fold enrichment
EL>5 EL<5
c
RNAP>2 RNAP<2
d
p-value<0.01
e
+TF -TF
f
MM -M - -
g
h
EL>5 RNAP>2
EL<5 RNAP<2
i
+TF M
-TF ~M
j
RNAP
EcTopoI
EcTopoI Rif+
EcTopoI CTD+
RNA-Seq
638 kb
639 kb
640 kb
641 kb
642 kb
4088 kb
4090 kb
4092 kb
4094 kb
4096 kb
4461 kb
4462 kb
4463 kb
4464 kb
4465 kb
4077 kb
4078 kb
4079 kb
4080 kb
dsbG
ahpC
ahpF
uspG
ybdR
malT
malP
malQ
gntT
gntY
yjfF
fbp
mpl
yjgA
pmbA
glgP
yzgL
glpD
glpE
glpG

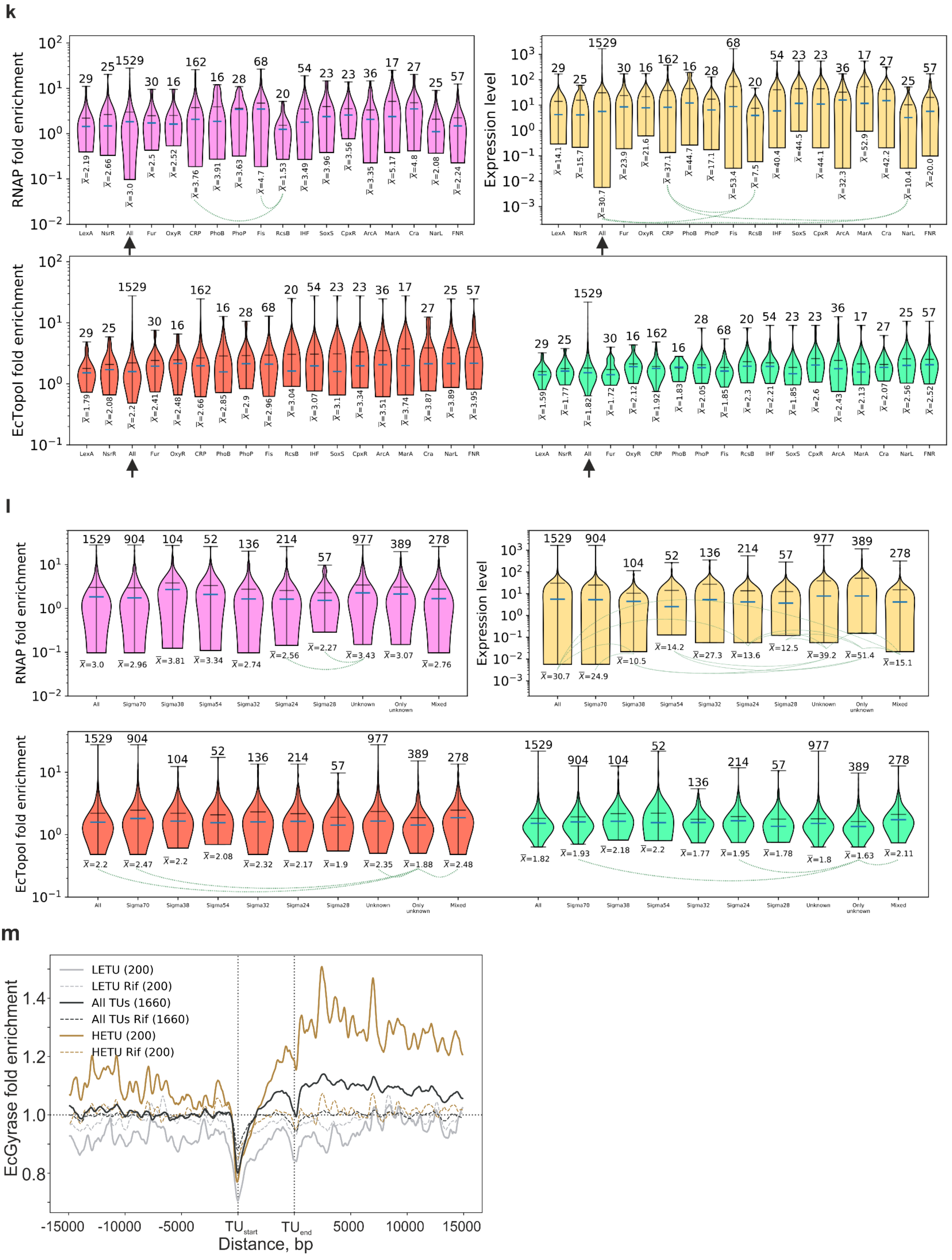

Supplementary Figure 6 (related to Figure 3). Factors associated with high or low EcTopoI enrichment signal at intergenic regions (IRs).

(**a**) Distribution of IR lengths in *E. coli* W3110 genome. In total, 3459 IRs were identified. For further analysis, regions were filtered by length (1000bp>x>50bp), and expression of adjacent genes (non-zero expression level). The *dps* gene and genes of rRNA operons were excluded due to the very high fold enrichment of EcTopoI at *dps* and the very high expression level of rRNA operons. Finally, 1529 IRs were analyzed.

(**b**) Effect of a high-level expression of adjacent genes. IRs were classified as regions associated with a high level of expression if the cumulative expression of adjacent genes was higher than 5 FPKM units (**EL>5**) and as regions associated with a low level of expression if the cumulative expression of adjacent genes was lower than 5 FPKM units (**EL<5**). Then, for each group of IRs, RNAP fold enrichment (**pink**), the expression level of adjacent genes (**yellow**), and fold enrichment of EcTopoI in Rif-/CTD- (**red**) and Rif+/CTD- (**green**) conditions were identified. Violin plots demonstrate the distribution of the parameter's values; the vertical axis is log-scaled. The sample size is indicated above the plots, means are marked with a horizontal black line and indicated below with ($\overline{X}$), medians are marked as a blue horizontal line.

(**c**) Violin plots showing the effect of RNAP with high and low enrichments at IR. IRs were classified as regions with a high RNAP signal if the fold enrichment was higher than 2 (**RNAP>2**) or as regions with a low RNAP signal if the fold enrichment was low (**RNAP<2**). The color coding and denotations are the same as in **b**.

(**d**) Violin plots for IRs classified by the orientation of adjacent genes: genes can be in the same orientation on the positive or negative strand (→→ or ←←), divergent (←→), or convergent (→←).

(**e**) Violin plots for IRs containing (**+TF**) and not containing (**-TF**) annotated binding sites for transcription factors (based on data from RegulonDB[2]).

(**f**) Violin plots for IRs classified by the presence of adjacent genes encoding membrane-localized protein: both genes encode membrane protein (**MM**), one gene encodes membrane protein (**M-**), and neither gene encodes membrane protein (--). The subcellular localization of *E. coli* proteins data was taken from EcoCyc database.

(**g**) Same analysis as in **F**, but the data on the subcellular localization of *E. coli* proteins was taken from PSORT database 4.0[3].

(**h**) Violin plots showing the combined effect of the two "expression-related" features – transcriptional activity and RNAP enrichment at IRs: high level of expression with high RNAP signal (**EL>5, RNAP>5**), and low expression with low RNAP signal (**EL<5, RNAP<2**).

(**i**) Violin plots showing the combined effect of three "expression-unrelated" features - orientation of adjacent genes (←→ or ~←→), the presence of transcription factor-binding sites (**+TF** or **-TF**), and adjacent genes encoding membrane-localized proteins (**M** or ~**M**). The data sets were taken from EcoCyc genome database, RegulonDB[2], and PSORT database 4.0[3], respectively.

(**j**) Examples showing the increased enrichment of EcTopoI in divergent IRs upstream of highly transcribed TUs and decreased enrichment in convergent IRs. Data for four representative genomic regions are shown. Enrichment of RNAP is shown in green; EcTopoI - in red; EcTopoI in Rif+ condition - in blue; EcTopoI in CTD+ condition - in magenta. RNA-Seq data are shown in grey. Data scaling is shown in parenthesis for each genomic track. Locations of IRs with specific enrichment patterns and directions of transcription of flanking genes is shown by black horizontal arrows.

(**k**) EcTopoI, RNAP enrichments and expression levels of genes flanking IRs of transcription factor site containing IRs classified by a type of a recognizing transcription factor. List of transcription factors considered: **LexA**, **Fur**, **NsrR**, **CRP**, **PhoP**, **OxyR**, **IHF**, **ArcA**, **RcsB**, **SoxS**, **PhoB**, **CpxR**, **Fis**, **NarL**, **FNR**, **MarA**, **Cra**. Only transcription factors with annotated sites in more than 15 IRs were considered. 1529 IRs were analyzed. For each set of IRs, RNAP fold enrichment (**pink**), the expression level of adjacent genes (**yellow**), and fold enrichment of EcTopoI under Rif-/CTD- (**red**) and Rif+/CTD- (**green**) conditions are shown. Violin plots demonstrate the distributions of the parameter's values; the vertical axis is in log-scale. The sample size is indicated above the plots; means are marked with a horizontal black line on the

plots and indicated below the plots with ($\overline{X}$), medians are marked as a blue horizontal line on plots. Annotation of transcription factor sites taken from RegulonDB[2]. Set comprising all intergenic regions is marked with a black arrow.

(**l**) EcTopoI, RNAP enrichments and expression levels of genes flanking IRs of promoter-containing IRs classified by a type of a recognizing sigma factor. 1529 IRs were analyzed. Depending on the annotated promoter recognized by specific sigma, the regions were classified as **Sigma70**, **Sigma38**, **Sigma54**, **Sigma32**, **Sigma24**, and **Sigma28**. If several different sigma factors recognize the same promoter, the region was marked as **Mixed**. If promoter specificity is unknown, the region was classified as **Unknown**. If all promoters annotated in a IR have an unknown specificity, the IR was classified as **Only unknown**. For each set of IRs, the RNAP fold enrichment (**pink**), the expression level of adjacent genes (**yellow**), and the fold enrichment of EcTopoI in Rif-/CTD- (**red**) and Rif+/CTD- (**green**) conditions were identified. The violin plots demonstrate the distribution of parameter values; the vertical axis is in log-scale. The sample size is indicated above the plots; means are marked with horizontal black line and indicated below with ($\overline{X}$), and medians are marked with blue horizontal line. Annotation of *E. coli* promoters was taken from RegulonDB[2].

For panels **b-i** differences between means were tested with two-sided Welch t-test. P-values lower than a threshold are indicated by asterisks, Bonferroni correction for multiple testing was applied: for panels **b**, **c**, **e**, **f**, **g**, **h**, **i** p-values<0.01 are indicated; for panel **d** p-values<8e-3 are indicated; for panel **k** p-values<3.3e-4 are indicated; for panel **l** p-values<1e-3 are indicated.

(**m**) Metagene plot showing DNA-gyrase enrichment in TUs, their upstream and downstream regions. Enrichment is shown for all TUs (black curve), highly-expressed (HETU, orange curve), and least-expressed (LETU, grey curve) sets. The number of TUs in each group is indicated in parentheses. DNA-gyrase data is from[4]; experiments with Cfx as a gyrase poison were used in the analysis. Data for gyrase under untreated and Rif-pretreated growth conditions are shown with solid and dashed curves, respectively. For all panels ChIP-Seq fold enrichment is given relative to the input sample.

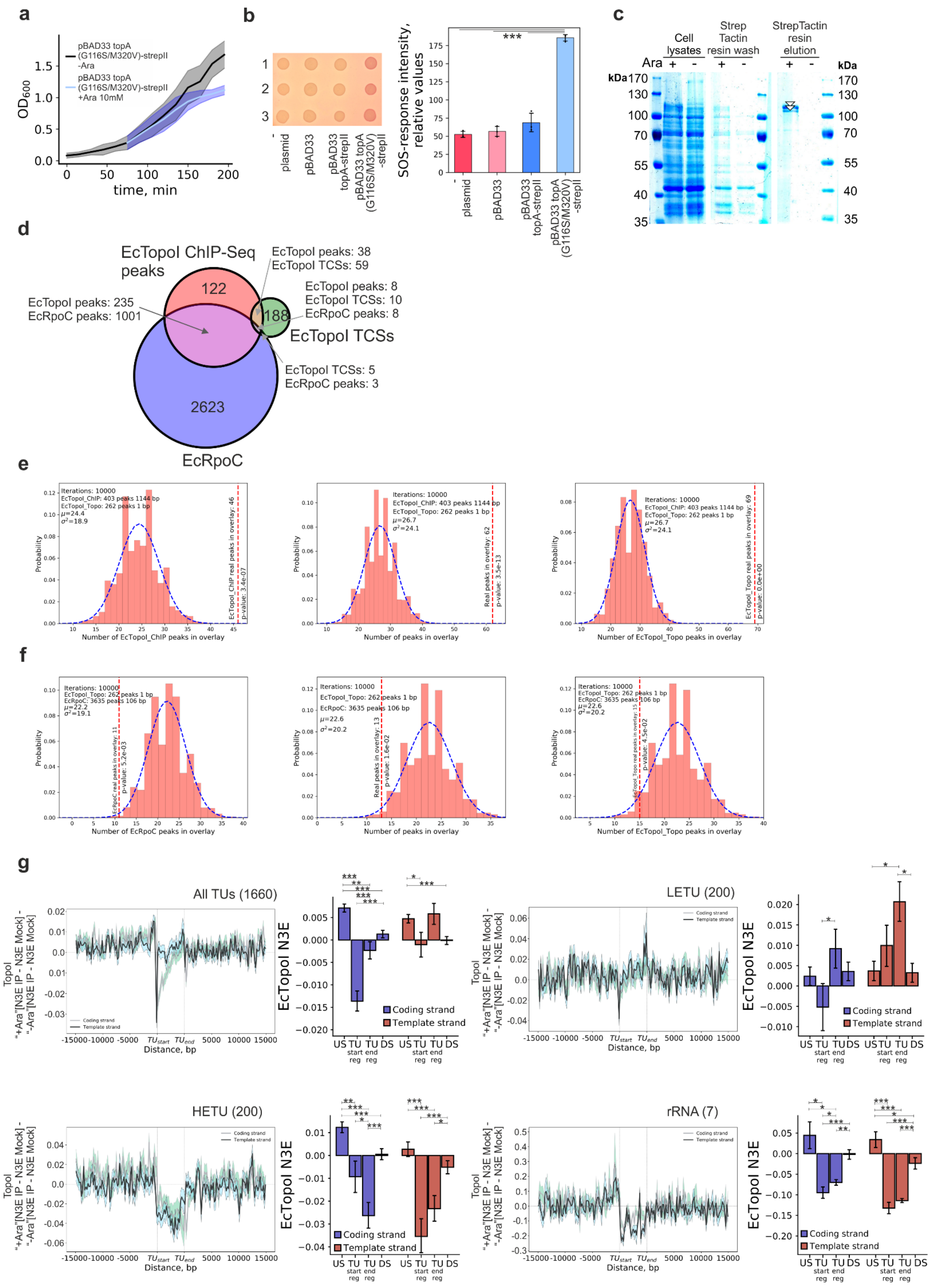
a
pBAD33 topA (G116S/M320V)-strepII -Ara
pBAD33 topA (G116S/M320V)-strepII +Ara 10mM
OD600
time, min
b
plasmid
pBAD33
pBAD33 topA-strepII
pBAD33 topA (G116S/M320V) -strepII
SOS-response intensity, relative values
***
c
Cell lysates
Strep Tactin resin wash
StrepTactin resin elution
Ara
kDa
d
EcTopoI ChIP-Seq peaks
122
EcTopoI peaks: 38
EcTopoI TCSs: 59
EcTopoI peaks: 8
EcTopoI TCSs: 10
EcRpoC peaks: 8
EcTopoI peaks: 235
EcRpoC peaks: 1001
188
EcTopoI TCSs
EcTopoI TCSs: 5
EcRpoC peaks: 3
2623
EcRpoC
e
Iterations: 10000
EcTopoI_ChIP: 403 peaks 1144 bp
EcTopoI_Topo: 262 peaks 1 bp
Number of EcTopoI_ChIP peaks in overlay
Number of peaks in overlay
Number of EcTopoI_Topo peaks in overlay
Probability
f
EcRpoC: 3635 peaks 106 bp
Number of EcRpoC peaks in overlay
g
All TUs (1660)
LETU (200)
HETU (200)
rRNA (7)
TopoI "+Ara"[N3E IP - N3E Mock] - "-Ara"[N3E IP - N3E Mock]
Distance, bp
EcTopoI N3E
Coding strand
Template strand
US TU start reg TU end reg DS

h

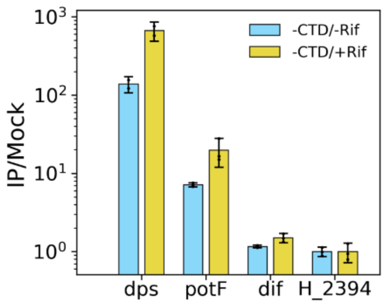

i

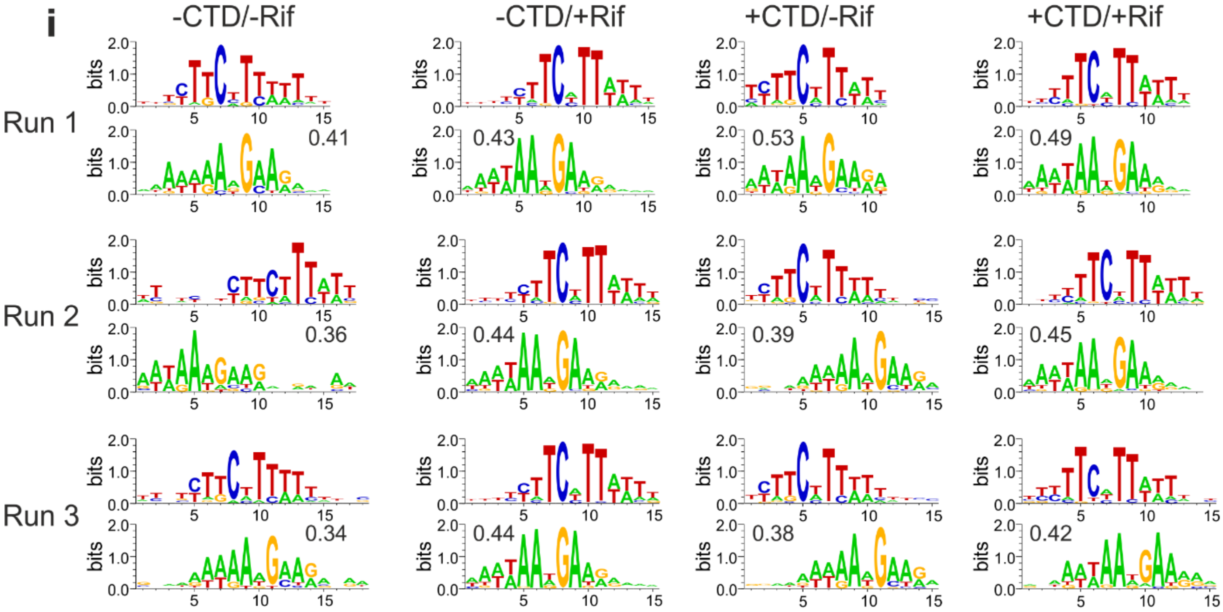

j

Experiment	Peaks (MACS2)	Peaks with motif (ChIPMunk)		
		Run1	Run2	Run3
-CTD/-Rif	403	363	379	382
-CTD/+Rif	327	320	287	284
+CTD/-Rif	445	420	439	445
+CTD/+Rif	242	228	242	240

k

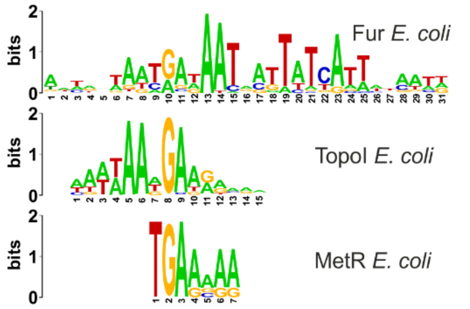

l

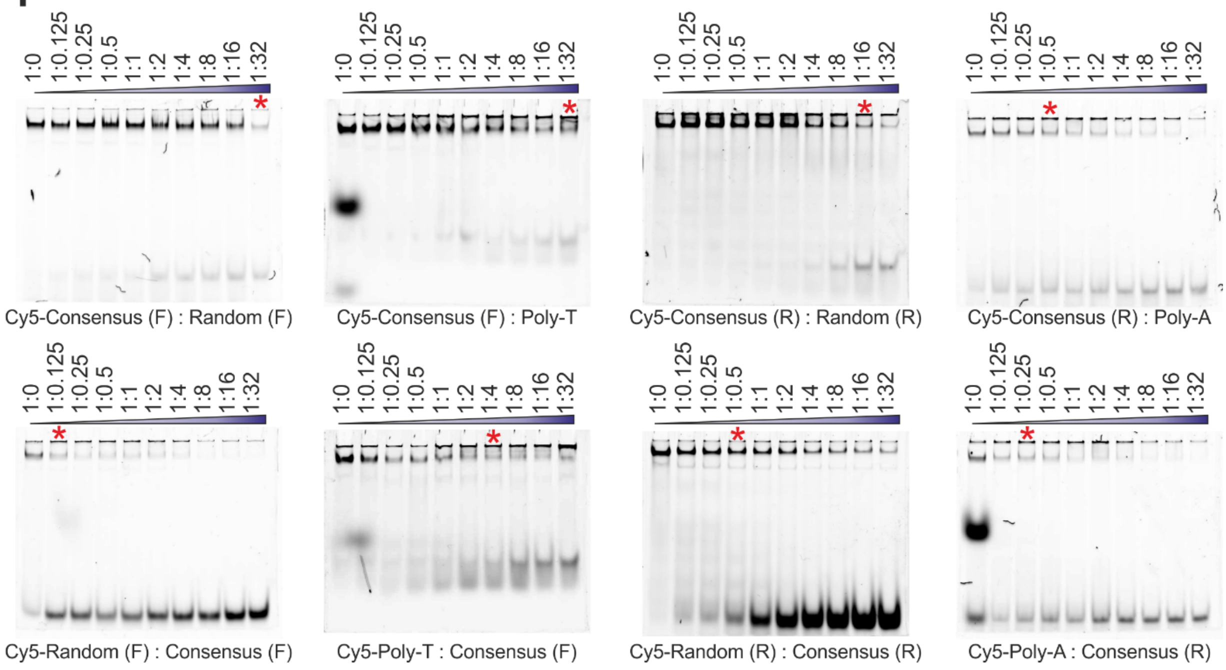

Supplementary Figure 7 (related to Figure 4). EcTopoI Topo-Seq and Topo-Seq data analysis.

(**a**) Growth curve of *E. coli* DY330 *topA-SPA* harboring pBAD33 topA(G116S/M320V)-strepII plasmid in LB supplemented with 0.5% glucose. Shadeed area represents 0.95 confidence intervals for the mean of three biological replicas. For details, see ***Supplementary Methods***. For source data, see **Table S8** in Source Data file.
(**b**) SOS-response is induced by EcTopoI G116S/M320V double-mutant overexpression from pBAD33 plasmid. *E. coli* CSH50 λ*sfiA::lacZ* reporter strains[5] (empty, harboring empty pBAD33, pBAD33 EcTopoI or pBAD33 EcTopoI G116S/M320V) were grown on MacConkey agar supplemented with 1% lactose and 1 mM Ara. SOS-response induction leads to *lacZ* expression, followed by active lactose digestion and acidification of the medium, which is monitored by color change – red color indicates acidification and SOS-response. Barplot on the right – qantification of SOS-response intensity (intensity of a red color adjusted for the background level). Differences in the signal were tested using the two-sided Welch t-test. P-values<2.5e-2 are indicated by asterisks with the number of asterisks indicating the significance level, Bonferroni correction for multiple testing was applied. Bars with error bars represent mean ±SD. The assay is shown for three biological replicates. For source data, see **Table S9** in Source Data file.
(**c**) Overexpression and purification of EcTopoI G116S/M320V from the pBAD33 plasmid during the Topo-Seq procedure (for details, see ***Methods***). Bands corresponding to EcTopoI G116S/M320V (verified by MS) are marked with white triangles. Experiment was performed in triplicate, a representative gel is shown.
(**d**) Correspondence between EcTopoI ChIP-Seq peaks, topoisomerase cleavage sites (TCSs) identified with Topo-Seq, and EcRpoC ChIP-Seq peaks. The Venn diagram demonstrates the number of overlapping peaks and TCSs.
(**e**), (**f**) Monte-Carlo simulation of the overlay between EcTopoI peaks and TCSs sets and between EcTopoI TCSs and EcRpoC peaks, respectively. Simulations were performed with the **Peak_overlap_simulation.py** script, 10000 simulations per test. For details, see ***Supplementary Methods***.
(**g**) Metagene plot representing the enrichment of EcTopoI cleavage activity (N3E) for All TUs set, LETU, HETU, and rRNA operons (rRNA). The number of TUs considered for each set is indicated in parentheses. To smooth the cleavage signal, it was binned using a bin width of 200 nt. For the cleavage tracks, confidential interval ±SEM is shown. Bar graphs show a comparison of EcTopoI cleavage signal in different regions relative to TUs. Differences in the signal were tested using the two-tailed Welch t-test. P-values<4e-3 are indicated by asterisks, Bonferroni correction for multiple testing was applied. Bars represent mean values ±SEM, n=200 TUs.
(**h**) ChIP-qPCR validation of ChIP-Seq peaks near the dps and potF genes. The dps and potF regions exhibit dramatically increased fold enrichment signals compared to the two control regions – dif and H2394. Bars with error bars represent mean enrichment ±SD. For source data, see **Table S10** in Source Data file. Experiment was repeated in three independent biological replicates.
(**i**) Binding motifs of EcTopoI identified in 3 independent runs of ChIPMunk for different ChIP-Seq experiments: -CTD/-Rif, -CTD/+Rif, +CTD/-Rif, +CTD/+Rif. Each motif is given for "+" and "-" strands. 3 independent runs were performed in accordance with the ChIPMunk manual because of the stochastic nature of the algorithm[6]. The significance of the motifs expressed as a Kullback Discrete Information Content value (KDIC) is indicated for each algorithm run.

(**j**) Number of peaks identified by MACS2[7] and used for motif discovery, and number of peaks containing motif identified by ChIPMunk.

(**k**) Motifs of *E. coli* DNA-binding proteins similar to EcTopoI motif identified by Tomtom[8]. Similarity was found between the EcTopoI motif and *E. coli* Fur (p-value 3.2e-3; e-value 1.6) and *E. coli* MetR (p-value 2.2e-3; e-value 1.1).

(**l**) EMSA competition experiments. Molar ratios between the labeled oligonucleotides and the non-labeled competitors are indicated above the gels. Oligonucleotide pairs are indicated below the gels (Cy5-labeled oligo: non-labeled oligo). The dye used to track the separation progression is visible as a black dot in the first lanes of several gels: Cy5-Consensus (F):poly-T and Cy5-Poly-A:Consensus (R). The minimal concentration, at which a non-labeled competitor starts to displace a labeled oligo, is indicated by a red asterisk. Experiments were performed in triplicate, representative gels are shown.

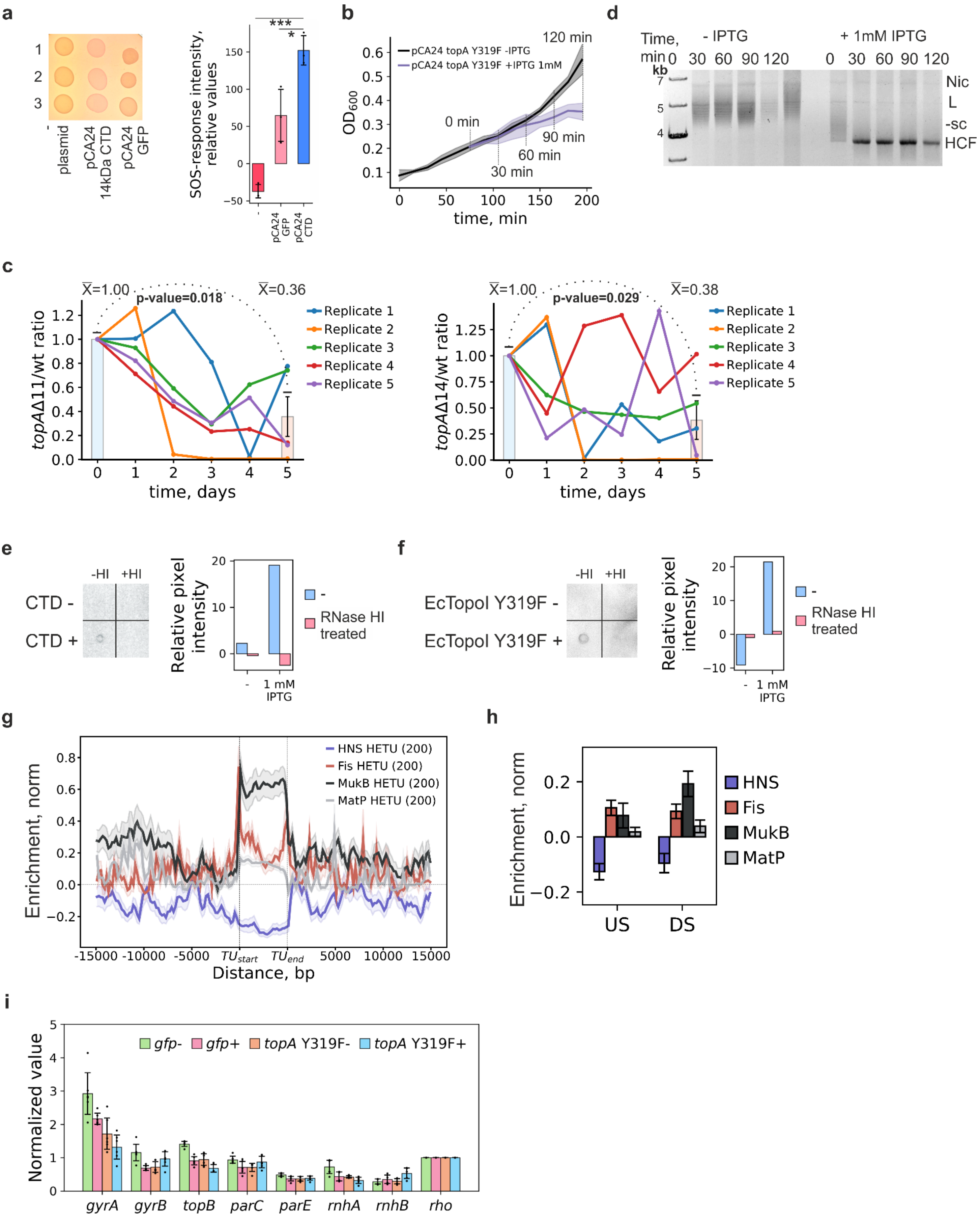

Supplementary Figure 8 (related to Figure 5). Physiological consequences of RNAP:EcTopoI complex disruption.

(**a**) SOS-respons is induced by EcTopoI 14kDa CTD overexpression. *E. coli* CSH50 λ*sfiA::lacZ* reporter strains[5] (empty, harboring pCA24 14kDa CTD or pCA24 GFP) were grown on MacConkey agar supplemented with 1% lactose. SOS-response induction leads to *lacZ* expression, followed by active lactose digestion and acidification of the medium, which is monitored by color change – red color indicates acidification and SOS-response. Barplot on the right – qantification of SOS-response intensity (intensity of a red color adjusted for the background level). Differences in the signal were tested using the two-sided Welch t-test. P-values<2.5e-2 are indicated by asterisks with the number of asterisks indicating the significance level, Bonferroni correction for multiple testing was applied. Bars with error bars represent mean ±SD. The assay is shown for three replicates. For source data, see **Table S13** in Source Data file.

(**b**) Growth curves of *E. coli* DY330 *topA*-SPA harboring pCA24 topA Y319F plasmid in LB supplemented with 0.5% glucose. At OD_{600}~0.2 1 mM IPTG was added to indicated cultures ("+ IPTG 1 mM", means of three biological replicates are shown), non-induced cultures served as controls ("- IPTG", means of three biological replicates shown). Shade represents 0.95 confidential interval of the mean. Grey lines mark time-points when aliquots were taken for plasmid extraction. For source data, see **Table S8** in Source Data file.

(**c**) Viability of *E. coli* BW25113 strains carrying truncated versions of a *topA* gene. Competition between BW25113 *wt* and BW25113 *topA* mutants (*topAΔ11* or *topAΔ14*). The mixture of strains was cultivated in LB and, after every 24h, inoculated into a fresh LB. Strain abundance was monitored with strain-specific primers using qPCR. Two-tailed Welch t-test was used to assess the differences between mutant/wt ratio at the beginning (day 0) and the end of the competition experiment (day 5). Bars and error bars represent mean ±SEM. Five independent biological replicates were done. For source data, see **Table S14** in Source Data file.

(**d**) Supercoiling of pCA24 topA Y319F plasmid extracted from exponentially growing *E. coli* DY330 *topA*-SPA cultures. At OD_{600}~0.2 cultures were either induced with IPTG ("+1 mM IPTG") or continued growth without induction ("- IPTG"). Cultures were sampled every 30 min, corresponding to time-points indicated in panel **b**. Nic – nicked plasmid, L – linear plasmid, -sc – negatively supercoiled plasmid, HCF – hypercompacted form of a plasmid. Plasmid forms were separated by electrophoresis in 1% agarose gels in TAE buffer supplemented with 5 µg/mL chloroquine and visualized by EtBr staining. Experiment was performed in triplicate; a representative gel is shown.

(**e**) Short-term (1 h**)** 14 kDa CTD overexpression from pCA24 14kDa CTD leads to R-loops accumulation. The dot-blot image is shown on the left; signal quantification is on the right. Samples were treated with RNase III to remove double-stranded RNA. For the dot-blot procedure, see ***Supplementary Methods***. For source data, see **Table S15** in Source Data file.

(**f**) Short-term (1 h**)** EcTopoI Y319F catalytically inactive mutant overexpression from pCA24 EcTopoI Y319F leads to R-loops accumulation. Legend as is for panel **e**. For source data, see **Table S15** in Source Data file.

(**g**) Metagene plot of normalized enrichment of NAPs (HNS - blue, Fis - red, MukB – black, MatP - grey) for HETUs. Confidence bands are represented by light-colored ±SEM profiles.

(**h**) Comparison of NAPs enrichment for HETUs (HNS - blue, Fis - red, MukB – black, MatP - grey). Enrichment was quantified for normalized tracks (from panel **g**) for 5 kb upstream regions (US) and 5 kb downstream regions (DS). Differences in enrichment between US and DS regions were tested using the two-sided Welch t-test. All P-values>5e-2. Bars represent mean values ±SEM, n=200 TUs for all conditions.

(**i**) qPCR quantification of transcription levels of supercoiling-related genes (*gyrA*, *gyrB*, *parC*, *parE*, *topB*, *rnhA*, *rnhB*) upon overexpression of *gfp* and *topA Y319F*. Expression levels are normalized relative to the transcription level of *rho*. For source data, see **Table S6** in Source Data file.

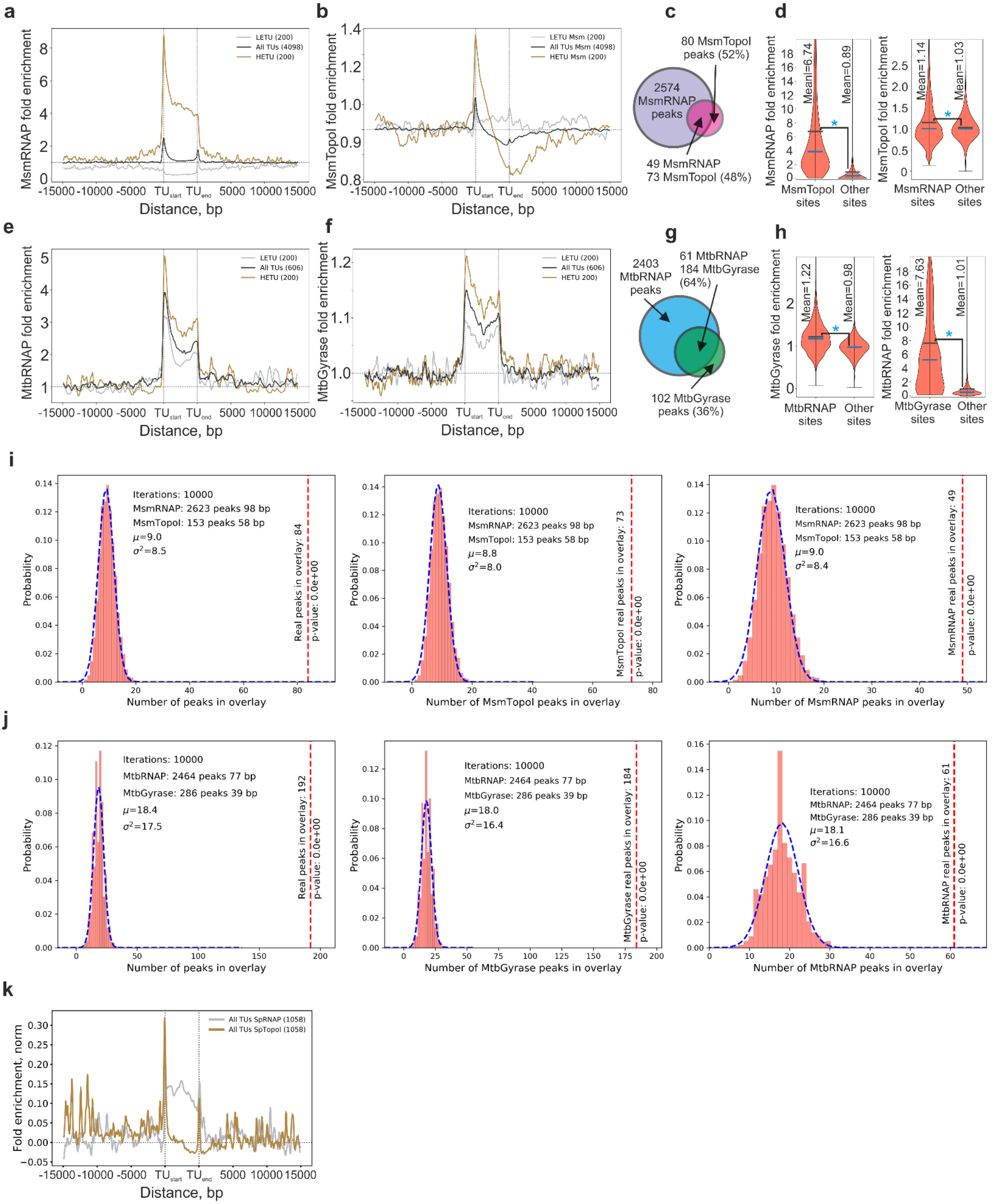

Supplementary Figure 9 (related to Figure 6). Interaction of TopoI and RNAP in *Mycobacteria* and variations of the twin-domain model.

(**a**) Metagene plot represents enrichment of MsmRNAP over HETU, LETU, and All TUs. The number of TUs considered for each set is indicated in parentheses. The transcription level of *M. smegmatis* TUs is assessed based on RNA-Seq data taken from[9], MsmRNAP ChIP-Seq data is taken from[10].

(**b**) Metagene plot represents enrichment of MsmTopoI over the TUs sets. MsmTopoI ChIP-Seq data is taken from[11].

(**c**) The Venn diagram represents the number of overlapping RNAP and TopoI peaks in *M. smegmatis*.

(**d**) Enrichment of MsmRNAP in regions occupied by MsmTopoI peaks and enrichment of MsmTopoI in regions occupied by MsmRNAP peaks. Mean and median are indicated by black and blue lines, respectively. The statistically significant differences between means (two-sided Welch t-test, p-value=8e-11 and 4e-21 for MsmRNAP and MsmTopoI enrichments, respectively) are indicated by asterisks.

(**e**) Metagene plot represents enrichment of MtbRNAP over HETU, LETU, and All TUs. The number of TUs considered for each set is indicated in parentheses. The transcription level of *M. tuberculosis* TUs is assessed based on RNA-Seq data taken from[12]. MtbRNAP ChIP-Seq data is taken from[12].

(**f**) Metagene plot represents enrichment of MtbGyrase over sets of TUs. MtbGyrase ChIP-Seq data is taken from[13].

(**g**) The Venn diagram represents the number of overlapping RNAP and gyrase peaks in *M. tuberculosis*.

(**h**) Enrichment of MtbGyrase in regions occupied by MtbRNAP peaks and enrichment of MtbRNAP in regions occupied by MtbGyrase peaks. Mean and median are indicated by black and blue lines, respectively. The statistically significant differences between means (two-sided Welch t-test, p-value=1e-92 and 4e-19 for MtbGyrase and MtbRNAP enrichments, respectively) are indicated by asterisks.

(**i**)-(**j**) Monte-Carlo simulation of peak sets overlay. Sampling the overlay of peak sets for MsmRNAP and MsmTopoI (**i**), MtbRNAP and MtbGyrase (**j**). Simulations were performed using the **Peak_overlap_simulation.py** script, 10000 simulations per test. For details, see ***Supplementary Methods***.

(**k**) Metagene plot represents enrichment of SpTopoI and SpRNAP over a set of all *S. pneumoniae* TUs. ChIP-Seq data is taken from[14]. For all panels ChIP-Seq fold enrichment is given relative to the input sample.

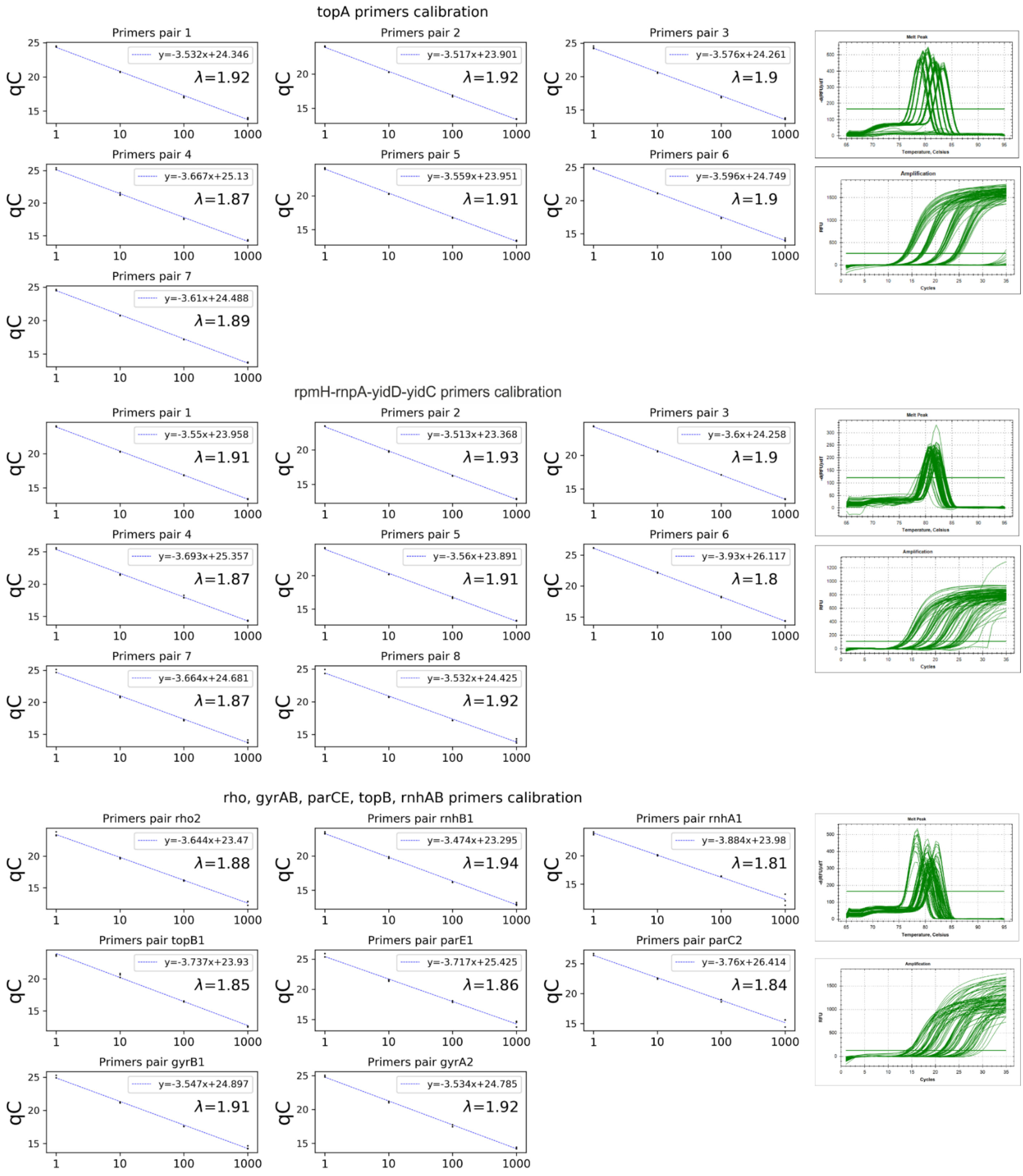

Supplementary Figure 10. Efficiency and specificity of primers used in ChIP-qPCR and RT-qPCR experiments. Calibration curves constructed by serial dilution of template DNA (*E. coli* genomic DNA): 1 – 0.075ng, 10 – 0.75ng, 100 – 7.5ng, 1000 – 75ng of template per reaction. Melting curves and amplification curves for groups of primers are shown on the right. For source data, see **Table S5** in Source Data file.

Supplementary Methods

Toxicity assay of EcTopoI G116S/M320V

E. coli DY330 *topA-SPA* strain harboring pBAD33 topA(G116S/M320V)-strepII plasmid was grown in LB supplemented with 0.5% glucose. At OD_{600}~0.2 the culture was divided and 10 mM Ara was added to one half (+Ara 10 mM, light blue curve), while the second half served as a non-induced control (-Ara, black curve). To construct growth curves, OD_{600} of the cultures were measured every 15 min. The experiment was performed in triplicate.

***E. coli* TopoI ChIP-Seq and DRIP-Seq**

Purification of IP-DNA with AMPure XP magnetic beads

To one volume of DNA solution one volume of binding buffer was added (10 mM Tris-HCl pH 8.0, 1 mM EDTA, 250 mM NaCl, 20% PEG-8000, 0.05% Tween 20) and one volume of AMPure XP beads. The suspension was incubated for 10 min at room temperature. Magnetic beads were separated on a magnetic stand and supernatant was discarded. Beads' pellet was carefully washed 3 times with 75% ethanol on a magnetic stand. Beads were dried and resuspended in 15 μL of deionized water. Beads were trapped on a magnetic stand and supernatant was collected and stored at -20°C.

***E. coli* RNAP ChIP-Seq**

For TAP-tagged RNAP-ChIP, we used *E. coli* DY330 strain (Dharmacon) carrying chromosomally TAP-tagged *rpoC* gene [15]. Cell culture (V=200 mL) grown to mid-exponential phase OD600~0.5-0.7 in LB containing 50 ug/mL of kanamycin was crosslinked by adding formaldehyde to a final concentration of 1%, followed by incubation for 20 min at room temperature, with agitation. Crosslinking was stopped by the addition of sterile glycine to a final concentration of 0.25M. Cells were incubated for 20 min at room temperature and harvested by centrifugation at 3000 x g, 10min, 4°C. Cell pellets were washed twice with 20 mL of ice-cold 20 mM Tris-HCl buffer pH 7.6, containing 60 mM NaCl (Tris-buffered saline, TBS), resuspended in 0.5 mL of 2x ChIP Lysis Buffer 1 (10 mM tris pH 8.0, 50 mM NaCl, 10 mM EDTA, 20% Sucrose), followed by addition of 0.5 mL of 2x ChIP Lysis Buffer 2 (200 mM Tris pH 8.0, 600 mM NaCl, 4% Triton X-100), containing RNase A (1 μg/mL). Resuspended cells were pre-lysed by the addition of 1 ug of Lysozyme and incubation at 37°C for 10 min, followed by chilling on ice. Next, cells were sonicated in a 2.0 mL Eppendorf tube in an ice-water bath, using microtip to achieve a high yield of 300-400bp fragments. Lysates were clarified by centrifugation at 13,000 x g for 10 min at 4°C, and the resulting supernatant was used for further analysis.

For the Input DNA fragment size analysis, a 100 μL aliquot of the clarified lysate was first treated with proteinase K (Thermo-Fisher Scientific, 25530049) at 0.125 mg/mL in the presence of 1%

SDS for 4 h at 37°C followed by DNA de-crosslinking by incubation at 65°C for 6 h. The Input DNA material was purified using the Qiagen PCR purification kit (Qiagen, 28104) and quantified by the Nano-Drop spectrophotometer. The DNA fragment size distribution (in 30-60 ng aliquots) was assessed by 8% DNA PAGE using Low Molecular Weight DNA markers (New England Biolabs, N3233S), visualized by SYBR-gold (Thermo fisher scientific, S11494) staining and quantified by the Kodak Imaging station.

For the preparation of RNAP-ChIP DNA, the remaining lysate (~900 μL) was purified by immunoprecipitation (IP). The lysate was mixed with 10 μL of IgG-agarose (GE Healthcare) and incubated at 4°C overnight on a rotating mixer in the presence of Protease Inhibitor Cocktail (Sigma, s8830).

Then affinity resin was washed consecutively with 1 mL each of the following solutions: 40 mM Tris pH 7.9, 0.5% Tween 20, 2 M NaCl; 40 mM Tris pH 7.9, 0.5% Tween 20, 1 M NaCl; 40 mM Tris pH 7.9, 0.5% Tween 20, 200 mM NaCl; and twice with RIPA buffer (50 mM Tris pH 7.4, 140 mM NaCl, 1% NP-40, 0.1% Deoxycholate, 0.1% SDS). All wash procedures were carried out by resuspending the beads in the wash solution and inverting the tube several times, followed by brief centrifugation and removing the supernatant by vacuum aspiration. The last wash was done for 20 min at 4°C on a rotary mixer. The RNAP-DNA crosslinks were eluted by incubation with ChIP Elution Buffer (10 mM Tris pH 8.0, 30 mM EDTA, 1% SDS) at 65°C for 4 hours (or O/N) on a shaker at 800-900 rpm. The resulting material was treated with Proteinase K (Thermo Fisher Scientific, 25530049) (0.2 mg/mL) at 37°C for 3-5h, followed by IP-DNA decrosslinking by incubation at 95°C for 2h. The IP-DNA was finally purified using the ChIP DNA Cleaning & Concentrator kit (Zymo Research, D5205).

A 50-100 ng of IP-DNA or Input-DNA was subjected to the following enzymatic and purification steps:

1. *End repair*

Reactions were performed in the final volume of 50 μL in the presence of a 1x T4 DNA ligase buffer (NEB, B0202s), 0.4 mM dNTP mix, 3 units of T4 DNA polymerase (NEB, M0203s), 10 units of T4 polynucleotide kinase (PNK) (NEB, M0201s), and 5 units of Klenow DNA polymerase (NEB, M0210s). Reactions were incubated for 30 min at 20°C, and DNA was purified by the Qiaquick PCR DNA purification kit (Qiagen, 28104) using 36 μL of Qiagen Elution Buffer (EB).

2. *Addition of "A" bases to the 3'-end of the DNA fragments*

The eluted DNA material from the previous step was combined with 0.2 mM dATP and 5 units of Klenow Fragment (3' to 5' exo minus) (NEB, M0212s) in 50 μL of Klenow buffer (NEB buffer 2). Reactions were incubated for 30 min at 37°C, and DNA was purified by the MinElute PCR purification Kit (Qiagen, 28004) using 15 μL of EB.

3. *Adapter ligation.*

Multiplex Adapter (MPA) mix was prepared by combining 1 umole of Illumina DNA adapters MP_Adapt1 and MP_Adapt2 with 1x NEB Buffer 2 in the final volume of 50 μL followed by incubation in a thermal cycler at 95°C for 5min, and then 80°C, 70°C, 60°C, 50°C, 40°C, and 30°C for 1 min each step. The MPA mix was diluted 1:20 in EB, to a final concentration of ~2 μM (needed for ligation). The DNA material from the previous step was used in a ligation reaction with 2 pmoles of diluted MPA oligo mix and 2000 units of the Quick DNA ligase (NEB, M2200s) in 30 μL of Quick DNA ligase Buffer (NEB, M2200s). Reactions were incubated for 15 min at 20°C, and DNA was purified by Agencourt Ampure XP beads (Beckman Coulter, A63880) using 11 μL of elution buffer.

4. *DNA size selection using 2% agarose gel electrophoresis*

Samples were separated on a 2% Low Range Ultra-Agarose gel (BioRad, 161-3106) in standard Tris-acetate-EDTA buffer (TEA) using Low Molecular Weight Ladder and stained by SYBR-gold DNA dye. DNA bands corresponding to 220 bp size were excised by Gel X-tracta tool (USA scientific, 5454-2500) and purified with the Gel Extraction Kit (Qiagen, 28704) using 38 μL of EB.

5. *PCR enrichment of the Adapter-Modified DNA fragments*

The purified DNA material was PCR-amplified using 2 uM PCR primer 1_1, 2 uM PCR primer 2_1 and 1 unit of Phusion polymerase (NEB, M0530s) in the presence 0.3mM dNTP in 1x Phusion HF buffer, under the following conditions: first cycle - 30 seconds at 98°C, followed by 18 cycles with 30 sec at 65°C and 30 sec at 72°C, completed by the final step for 5 minutes at 72°C. The resulting PCR-amplified DNA was purified by the MinElute PCR purification kit (Qiagen, 28004) using 16 μL of EB.

Input and IP DNA samples were analyzed by Nano-Drop spectrophotometer and 8% DNA-PAGE with SYBR-gold (Thermo Fisher scientific, S11494) staining quantified by the Kodak Gel Logic 200 Imager. The final library DNA concentrations were analyzed by qPCR using the KAPA Illumina library quantification kit (Kapa Biosystems, KK4824).

DNA sequencing was performed by Illumina HiSeq 50 bp paired-end conditions using 24 TruSeq Index Primers (Illumina).

De-multiplexed and de-barcoded, QC-filtered short-read sequences were aligned to the *E. coli* W3110 MuSGS genome based on NC_007779.1 sequence (https://github.com/sutormin94/TopoA_ChIP-Seq/blob/master/Additional_genome_features/E_coli_w3110_G_Mu.fasta) using Bowtie v 1.2.2 for Illumina short read aligner tool[16]. Mapping statistics, read depth, and ChIP signal quality assessment was calculated using DeepTools v2.5.0[17].

Peak calling, signal normalization, and PCR duplicate removal were performed using MACS2 v2.2.6[18]. Fold enrichment of RNAP ChIP signal over Input signal was performed using bdgcmp ("BedGraph Compare") function of MACS2 using Fold Enrichment (FE) method. The resultant signal tracks represented normalized binding profiles were visualized using IGV v2.7.2[19]. Fold enrichment track was used for further analysis using custom python scripts (https://github.com/sutormin94/TopoA_ChIP-Seq).

***Mycobacterium tuberculosis* ChIP-Seq (MtbRNAP, MtbGyrase) and RNA-Seq data analysis**

The expression level of *M. tuberculosis* TUs in a log-phase was taken from Uplekar et al., 2013 (Supplementary material, Table 7)[12]. Top 200 of 606 TUs by expression level comprise HETU set (highly-expressed TUs, FPKM > 4.75), similarly 606 TUs with the lowest expression level comprise LETU set (least-expressed TUs, FPKM < 1.9).

The publicly available RNAP β-subunit ChIP-Seq datasets for exponentially growing culture of *M. tuberculosis* H37Rv were taken from GEO (GSM1003214, GSM1003215)[12]. Raw reads were trimmed and filtered using Trimmomatic and aligned to the *M. tuberculosis* H37Rv genome (NC_000962.3) using BWA-MEM[20]. PCR duplicates were not removed, because the dataset was comprised of single-stranded reads. Coverage depth bed track was obtained using samtools and converted to wig format with a custom script (**MtbRNAP IP tracks**). Mock control datasets from the same study (GSM1003222, GSM1003223) were processed identically (**Mock tracks**). Fold enrichment tracks for RNAP β-subunit of *M. tuberculosis* were obtained as a position-wise ratio of normalized **IP tracks** and corresponding **Mock tracks**. Fold enrichment tracks of biological replicates (GSM1003214/GSM1003222, GSM1003215/GSM1003223) were position-wise averaged. Regions of a genome having fold enrichment > 3 were defined as MtbRNAP peaks. Totally, 2464 peaks were identified.

The publicly available DNA-gyrase ChIP-Seq dataset for exponentially growing culture of *M. tuberculosis* H37Rv was taken from GEO (GSM2538162)[13]. Raw reads were trimmed and filtered

using Trimmomatic and aligned to the *M. tuberculosis* H37Rv genome (NC_000962.3) using BWA-MEM[20]. PCR duplicates were removed, using samtools. Coverage depth bed track was obtained using samtools and converted to wig format with a custom script (**MtbGyrase IP track**). Mock control dataset from the same study (GSM2538163) was processed identically (**Mock track**). Fold enrichment track for DNA-gyrase of *M. tuberculosis* was obtained as a position-wise ratio of normalized **IP track** and a **Mock track**. Regions of a genome having fold enrichment > 2 were defined as MtbGyrase peaks. Totally, 286 peaks were identified.

Mycobacterium smegmatis **ChIP-Seq (MsmRNAP, MsmTopoI) and RNA-Seq data analysis**

The publicly available RNA-Seq datasets for a mid-exponential culture of *M. smegmatis* were taken from GEO (GSM2756262, GSM2756263) for estimation of the expression level of TUs[9]. Raw reads were trimmed and filtered using Trimmomatic and aligned to the *M. smegmatis* MC^2*-155* genome (CP009496.1) using BWA-MEM[20]. PCR-duplicates were removed with samtools [21]. Genome annotation and TUs coordinates were taken from BioCyc[22] for genome assembly NC_008596.1 and transferred for CP009496.1 using blast. The expression levels of TUs were calculated as FPKM value using FPKM_count.py from RSeQC package[23]. Final TUs FPKMs were calculated as a mean of two biological replicates (GSM2756262, GSM2756263). Top 200 of 4098 TUs by expression level comprise HETU set (highly-expressed TUs, FPKM > 634.8), similarly, 200 TUs with the lowest expression level comprise LETU set (least-expressed TUs, FPKM < 3.2).

The publicly available RNAP β-subunit ChIP-Seq datasets for exponentially growing culture of *M. smegmatis* were taken from GEO (GSM1171544, GSM1171545)[10]. Raw reads were trimmed and filtered using Trimmomatic and aligned to the *M. smegmatis* MC^2*-155* genome (CP009496.1) using BWA-MEM [20]. PCR duplicates were not removed, because the datasets comprised of single-stranded reads. Coverage depth bed tracks were obtained using samtools and converted to wig format with a custom script. Tracks of biological replicates (GSM1171544, GSM1171545) were position-wise averaged (**MsmRNAP IP track**).

Mock control datasets for *M. smegmatis* ChIP-Seq experiments were taken from GEO (GSM4274349, GSM4274350) [24]. Raw reads were trimmed and filtered using Trimmomatic and aligned to the *M. smegmatis* MC^2*-155* genome (CP009496.1) using BWA-MEM[20]. PCR-duplicates were removed with samtools[21]. Coverage depth bed tracks were obtained using samtools and converted to wig with the custom script. Tracks of biological replicates (GSM4274349, GSM4274350) were position-wise averaged (**Mock track**).

Fold enrichment track for RNAP β-subunit of *M. smegmatis* was obtained as a position-wise ratio of normalized **MsmRNAP IP track** and **Mock track**. Regions of a genome having fold enrichment > 4 were defined as MsmRNAP peaks. Totally, 2623 peaks were identified.

The publicly available TopoI ChIP-Seq dataset for exponentially growing culture of *M. smegmatis* was taken from SRA (BioSample SAMN10371555, SRA ID SRR8149480)[11]. Raw reads were trimmed and filtered using Trimmomatic and aligned to the *M. smegmatis* MC^2*-155* genome (CP009496.1) using BWA-MEM[20]. PCR duplicates were removed with samtools[21]. Coverage depth bed track was obtained using samtools and converted to wig with the custom script (**MsmTopoI IP track**). Fold enrichment track for TopoI of *M. smegmatis* was obtained as a position-wise ratio of normalized **MsmTopoI IP track** and **Mock track**. Regions of a genome having fold enrichment > 3 were defined as MsmTopoI peaks. Totally, 153 peaks were identified.

E. coli GapR-Seq data analysis

The publicly available GapR-Seq datasets for *E. coli* MG1655 in exponential phase were taken from GEO (GSM4628314, GSM4628313, GSM4628312, GSM4628311) for mapping of positive and negative supercoiling over the *E. coli* genome[25]. Raw reads were trimmed and filtered using Trimmomatic and aligned to the *E. coli* W3110 MuSGS genome (*E. coli* W3110 genome with the insertion of *cat*-Mu SGS cassette may be downloaded from GEO: GSE95567) using BWA-MEM[20]. BAM and bed files were prepared with Samtools[21] and visualized in IGV[19]. Biological replicates for MG1655 pKVS45-GapR experiments (GSM4628312, GSM4628311) and MG1655 pKVS45-GapR-3xFLAG experiments (GSM4628314, GSM4628313) were scaled by the total amount of mapped reads and averaged separately. Fold enrichment tracks were prepared by the position-wise division of GapR-FLAG resultant track by GapR track (see https://github.com/sutormin94/TopoA_ChIP-Seq).

E. coli RpoC ChIP-chip data analysis

Publicly available RpoC ChIP-chip datasets for *E. coli* MG1655 treated with RNAP inhibitor rifampicin in exponential phase were taken from GEO (GSM351003) for mapping of RNAP enrichment over the *E. coli* genome[26]. The coordinates of the microarray oligonucleotides were identified in reference *E. coli* W3110 MuSGS genome (*E. coli* W3110 genome with the insertion of *cat*-MuSGS cassette may be downloaded from GEO: GSE95567) using blast. For each genomic position, a fold enrichment was calculated as an average enrichment of oligonucleotides aligned to this position (https://github.com/sutormin94/TopoA_ChIP-Seq/blob/master/Convert_chip_oligos_to_coordinates_Mooney_data.py).

Meta-gene analysis

To produce scaled meta-gene plots, fold enrichment of a protein of interest or another signal (coverage depth, GC%, etc.) was extracted in vicinity of transcription units (15 kb upstream, TU body, 15 kb downstream) in concordance to their orientation. Regions were scaled to have the

same number of positions (5000 bp) by omitting of randomly chosen points (if a region is longer than 5000) or by random duplication of points (if a region is shorter than 5000). Data extraction and scaling were performed with *FE_over_US_GB_DS.py* custom script. The resulting scaled arrays were averaged by position, smoothed with averaging sliding window 200bp and plotted with *Plot_signal_over_transcription_units.py* custom script. Zoom-in meta-gene plots representing the proximity of transcription start and transcription and sites (+300 bp:-200 bp and -200 bp:+300 bp, respectively) were produced without smoothing.

Normalization of ChIP-Seq and Topo-Seq data for the construction of joint metagene plots with several types of data or with data from different experiments was performed as following. For each nucleotide position of a metagene plot the z-score transformation was applied – a genome average was subtracted from the signal values and the result was divided by a genome standard deviation.

DRIP-Seq data was analyzed similarly, but in respect to the data strand-specificity, the signals for TUs in a reverse orientation were multiplied by -1 to flip them and make them concordant with signals for TUs in forward orientation (see *FE_over_US_GB_DS_str_spec.py* script in the https://github.com/sutormin94/E_coli_DRIP-Seq_analysis repository). For Topo-Seq data, in respect to the data strands-specificity, signals for coding and template strands of TUs in a forward orientation were taken from N3E_F and N3E_R tracks respectively; signals for coding and template strands of TUs in a reverse orientation were taken from N3E_R and N3E_F tracks respectively (see *FE_over_US_GB_DS_strand_specific_binning_and_statistics.py* script in the https://github.com/sutormin94/TopoI_Topo-Seq repository).

Identification of EcTopoI cleavage sites (TCSs)

Separately for forward and reverse strands, the number of DNA fragments 3'-ends was calculated per position (N3E) based on read alignments stored in SAM files (giving N3E_F and N3E_R tracks respectively). The tracks were scaled by the total number of aligned reads to get normalized coverage across samples and biological replicates were averaged. After that -IP tracks (+Ara-IP and -Ara-IP) were subtracted from corresponding +IP tracks (+Ara+IP and -Ara+IP respectively) strand-wise resulting in +Ara and -Ara tracks. TCSs were detected as sites having a signal higher than threshold value 15 (values 10 and 20 were also tested and given similar results in respect to identified DNA motif).

Sampling the colocalization of ChIP-Seq peaks using the Monte-Carlo simulation

Simulations were performed using the **Peak_overlap_simulation.py** script. Assume, the colocalization of peaks from set A and set B is assessed. First, protein-occupied regions (peaks) comprising the set A are randomly (genomic positions are uniformly sampled, but not allowing

the peak overlapping) placed in the genome. Similarly, and independently of set A placement, set B peaks are randomly placed. Finally, the number of overlapping peak pairs of which one peak belongs to set A and another peak belongs to set B is calculated and keeped. Indipendent simulations are repeated 10000 times per test and the distribution of resultant numbers is approximated with the normal distribution. Using the probability distibution, the real number of overlaps between set A and set B peaks observed in the initial experiment is statistically assesed.

Purification of EcTopoI

15 mL of a night culture of *E. coli* BL21(DE3) pET28 topA_strep in LB, supplemented with kanamycin 50 μg/mL, was added to 1.5 L of LB medium and cultivated at 37°C and 180 rpm. At OD_{600}~0.6 the culture was induced with IPTG (final concentration 0.4 mM) and cultivation continued for 4 h. The cells were pelleted (5000 rpm, 10 min, 4°C) and frozen in liquid nitrogen. Subsequently, the pellet was thawed on ice and resuspended in 5 mL of Lysis Buffer (100 mM Tris HCl pH 8.0, 150 mM NaCl, 1 mM EDTA) supplemented with 1 mM PMSF, lysed using incubation with 2 mg/mL lysozyme (1 h, 4°C) followed by sonication for 10 min. The obtained lysate was cleared by centrifugation (14000 rpm, 20 min, 4°C). Strep-tagged EcTopoI was purified on StrepTrap HP column (GE Healthcare). Briefly, the lysate was loaded on the 5 mL column pre-equilibrated with Lysis Buffer and the column was washed with 50 mL of Lysis buffer. Then proteins bound were eluted using 10 mL of Elution Buffer (50 mM Tris HCl, pH 8.0, 150 mM NaCl, 1 mM EDTA, 2.5 mM desthiobiotin). The eluted proteins were analyzed using electrophoresis in 11% PAA gel, the bands were visualized with Instant Blue protein stain and subjected to protein identification via MS. 10 mL of the eluate was dialyzed overnight at 4°C against 1.5 L of the Storage buffer (10 mM Tris-HCl pH 7.5, 50 mM KCl, 0.1 mM EDTA, 1 mM 2-mercaptoethanol) and then concentrated 5x on Amicon Ultra-15 filter (Millipore) by centrifugation (3500 g, 20 min, 4°C). Glycerol was added to 30% w/v and the sample was stored at -20°C. The activity of the enzyme was confirmed by relaxation assay with a negatively supercoiled pBR322 plasmid.

Relaxation assay with EcTopoI

2 μL (330 ng/μL) of negatively supercoiled pMP1000 plasmid extracted from *E. coli* DH5α cells was combined with 2 μL of 10x Reaction buffer (100 mM Tris-HCl pH 8.0, 500 mM NaCl, 60 mM $MgCl_2$), different amount of EcTopoI (0, 50, 100, 200, 300, 400 ng of the enzyme with concentration 170 ng/μL), and milli-Q H_2O up to 20 μL. The mixture was incubated 30 min at 37°C and the reaction was stopped by the addition of 5 μL of 5x Stop-buffer (50 mM EDTA pH 8.0, 0.5% bromophenol blue, 50% glycerol). Relaxation products were separated by

electrophoresis in 1% agarose gel supplemented with 2 μg/mL chloroquine. After separation, for DNA visualization gel was stained with ethidium bromide.

Protein affinity purification

To demonstrate that the expression of the EcTopoI 14kDa CTD fragment was induced in the samples used for physiological experiments upon the addition of IPTG, we performed a small-scale affinity purification of N-terminally 6His-tagged protein from cell lysate with subsequent SDS-PAGE analysis and MS-analysis.

Cell culture (100 mL) of *E. coli* DY330 *topA*-SPA pCA24 14kDa CTD was started by the addition of 1 mL of the corresponding overnight culture to fresh LB medium supplemented with 50 μg/mL kanamycin, 34 μg/mL chloramphenicol, and 0.5% glucose. After reaching the OD_{600}~0.2 the culture was bisected and one half of it was induced with 1 mM IPTG, the other was used as a control. The cells were pelleted (5000 rpm, 10 min, 4°C) after 1 h of cultivation and frozen in liquid N_2. Subsequently, each cell pellet was thawed on ice and resuspended in 1.5 mL of Lysis Buffer (50 mM Tris HCl pH 8.0, 150 mM NaCl, 2.5 mM Imidazole), lysed using incubation with 2 mg/mL lysozyme (1 h, 4°C) followed by sonication for 10 min. The obtained lysate was cleared by centrifugation (14000 rpm, 20 min, 4°C) and added to 30 μl of TALON superflow affinity resin (Sigma) pre-equilibrated with Lysis Buffer. After 1 h of incubation at 4°C with constant rotation the resin was washed five times with Lysis Buffer, the proteins bound were eluted using 40 μl of Elution Buffer (50 mM Tris HCl pH 8.0, 150 mM NaCl, 300 mM Imidazole). The eluted proteins were analyzed using electrophoresis in 11% PAA gel, the bands were visualized with Instant Blue protein stain and subjected to protein identification via MS.

Protein identification by MS

Small pieces of the bands of interest were cut out from the gel, washed from the stain twice with 60 mM $(NH_4)_2CO_3$ in 40% acetonitrile and dehydrated with 100% acetonitrile. After the addition of the digestion mixture containing 50 mM $(NH_4)_2CO_3$ and 15 ng/μL sequencing grade trypsin, the samples were incubated overnight at 37°C. The peptides were extracted using 10 μl of 0.5% trifluoroacetic acid. 2-3 μl of the obtained sample were subjected to MS analysis on UltrafleXtreme MALDI-TOF-TOF mass spectrometer (Bruker Daltonics) to get the peptide mass fingerprint. NCBI protein database search with Mascot software (Matrix Science) was performed for protein identification.

Pull-down of proteins bound to EcTopoI

An isolated colony of *E. coli* DY330 *topA-SPA* was inoculated in 5 mL of LB and cultivated overnight at 37°C with shaking 180 rpm. The next day the starter was diluted 100x with fresh

2xYT to get 200 mL of culture and cultivated at 37ºC until reaching OD_{600}~0.6-0.7. Then the culture was cooled on ice and centrifuged at 4ºC, 5000 rpm for 15 min. Cells pellet was frozen in liquid N_2 and stored at -80ºC. Then the pellet was dissolved in 3 mL of PBS with the addition of Proteoblock cOmplete and lysozyme (2 mg/mL) and incubated for 1 h at 25ºC. Cells were gently lysed by 2 repetitive cycles of freeze-thawing and then sonicated (5s pulse/5s relaxation for 2 min, 50% power, SONOPULS HD 3100). Cells debris was removed by centrifugation at 4ºC, 15000 rpm for 15 min and cleared lysate was incubated with 100 µL of ANTI-FLAG® M2 affinity gel (Sigma-Aldrich) at 4ºC for 1 h with constant rotation. Affinity resin was washed 4 times with 1 mL of PBS and bound proteins were eluted by 10 min incubation at 95ºC with 30 µL of 3x Laemmli buffer supplemented with 2-mercaptoethanol. Proteins were separated in 10% PAAG and were identified by MS.

Pull-down experiments with *E. coli* DY330 *topA-SPA* transformed with a plasmid (pCA24 GFP or pCA24 14kDa CTD) were performed essentially as described above. The starter culture was supplemented with 0.5% glucose and 34 µg/mL chloramphenicol and grown overnight. Next day, the starter was diluted 100x with 2xYT supplemented with glucose and antibiotic to get 400 mL of culture. The culture was cultivated until OD_{600}~0.2 and then bisected. One-half of it was induced with 1 mM IPTG and the cultures were cultivated for 1 h until OD_{600}~0.6-0.7.

Pull-down of proteins bound to EcTopoI 14kDa CTD and analysis by Western-blot

Cell cultures (100 mL) of *E. coli* DY330 *topA*-SPA pCA24 14kDa CTD or *E. coli* DY330 *rpoC*-TAP pCA24 14kDa CTD were started by the addition of 1 mL of the corresponding overnight culture to fresh LB medium supplemented with 50 µg/mL kanamycin, 34 µg/mL chloramphenicol, and 0.5% glucose. After reaching the OD_{600}~0.2 the culture was bisected and one half of it was induced with 1 mM IPTG, the other was used as a control. The cells were pelleted (5000 rpm, 10 min, 4ºC) after 1 h of cultivation and frozen in liquid N_2. Subsequently, each cell pellet was thawed on ice and resuspended in 1.5 mL of Lysis Buffer (50 mM Tris HCl pH 8.0, 150 mM NaCl, 5 mM Imidazole) with the addition of Proteoblock cOmplete, and lysed by sonication for 10 min. The total concentration of proteins in the obtained lysates was measured by Bradford assay (Protein Assay Dye Reagent Concentrate, Bio-Rad). Equal amounts of lysates (by total protein amount) were added to 80 µL aliquots of TALON superflow affinity resin (Sigma) pre-equilibrated with Lysis Buffer. After over-night incubation at 4ºC with constant rotation the resin was washed five times with 1 mL of Lysis Buffer, the proteins bound were eluted using incubation with 30 µL of Elution Buffer (50 mM Tris HCl pH 8.0, 150 mM NaCl, 300 mM Imidazole) for 15 min at room temperature. Not-bound fractions and the eluted proteins were analyzed using electrophoresis in 10% PAA gel, the bands were visualized with Instant Blue protein stain and subjected to protein identification via MS.

To specifically detect SPA-tagged EcTopoI (for *E. coli* DY330 *topA-SPA* pCA24 14kDa CTD) and TAP-tagged RpoC (for *E. coli* DY330 *rpoC-TAP* pCA24 14kDa CTD) among proteins co-purified with EcTopoI 14kDa CTD a Western blot was performed.

Not-bound fractions and the eluted proteins were analyzed using electrophoresis in 10% PAA gel and proteins were transferred to Hybond-P membrane (Amersham) using Trans-Blot SD Semi-dry transfer cell (Bio-Rad). Then the membrane was blocked with 25 mL of TBST buffer (20 mM Tris-HCl pH 7.5, 150 mM NaCl, 0.1% Tween 20) + 5% milk for 30 min with agitation 50 rpm on an orbital shaker. The incubation and all the subsequent incubations were performed at room temperature. Then the membrane was washed 3 times for 5 min with 25 mL TBST. Primary anti-FLAG antibodies produced in rabbits (Sigma Aldrich, F7425) were added 1:10000 in 25 mL of TBST and hybridized with the membrane for 1.5 h. Unbound antibodies were washed out with 25 mL TBST (3 times, 5 min each). Secondary anti-rabbit antibodies produced in goat conjugated with HRP (Sigma Aldrich, A0545) were added 1:25000 to 25 mL of TBST + 5% milk and hybridized with the membrane for 1 h. Not bound antibodies were washed out with 25 mL of TBST (3 times, 5 min each). Signal of HRP was detected using Clarity Western ECL Substrate (Bio-Rad) and captured with Fusion Solo S detection system (Vilber Lourmat).

Dot-blot with S9.6 antibodies against RNA:DNA hybrids

A single colony of *E. coli* DY330 *topA-SPA* harboring pCA24 14kDa CTD was inoculated into 5 mL of LB supplemented with chloramphenicol 34 μg/mL and 0.5% glucose and grown overnight. The starter (1 mL) was diluted 100 times with 100 mL of LB containing the antibiotic and 0.5% glucose and cultivated until OD_{600}~0.2 at 37°C with shacking, then the culture was bisected, and one half was induced with 1mM IPTG and cultivated for 1 h. Cells were harvested by centrifugation and the total nucleic acids were purified using the GeneJET Genomic DNA purification kit (ThermoFisher) according to the manufacturer protocol but omitting the RNase A treatment. Aliquots containing 100 ng of DNA (concentration measured by the Qubit using a dsDNA HS assay kit, Invitrogen) were treated with 1U of RNase III (Ambion) or with 1U of RNAase III and 5U of RNase HI (NEB) for 2h at 37°C in RNase III reaction buffer (10 mM Tris-HCl pH 7.9, 50 mM NaCl, 10 mM $MgCl_2$, 1 mM DTT) and nucleic acids were purified with the GeneJET Gel Extraction & DNA cleanup micro kit (General cleanup protocol, ThermoFisher). Equal amounts of samples (RNase III or RNase III/RNase HI treated) equivalent to 50 ng of DNA (concentration measured by the Qubit using dsDNA HS assay kit, Invitrogen) were applied onto the Hybond-N+ membrane (Amersham) and air-dried. The membrane was crosslinked with UV (70000 μJoules/cm^2) using HL2000 Hybrylinker. Then, the membrane was blocked with 25 mL of TBST buffer (20 mM Tris-HCl pH 7.5, 150 mM NaCl, 0.1% Tween 20) + 5% milk for 30 min with agitation 50 rpm on an orbital shaker. The incubation and all the subsequent incubations were

performed at room temperature. Then, the membrane was washed 3 times for 5 min with 25 mL TBST. 15 µg (1:1850) of primary S9.6 antibodies produced in mice (Kerafast, ENH001) were hybridized with the membrane in 25 mL of TBST for 1.5 h. Unbound antibodies were washed out with 25 mL TBST (3 times, 5 min each). Secondary anti-mouse antibodies conjugated with HRP (Sigma Aldrich, A9044) were added 1:80000 to 25 mL of TBST + 5% milk and hybridized with the membrane for 1 h. Not bound antibodies were washed out with 25 mL of TBST (3 times, 5 min each). Signal of HRP was detected using Clarity Western ECL Substrate (Bio-Rad) and captured with Fusion Solo S detection system (Vilber Lourmat).

Assessing the expression level of genes by RT-qPCR in response to *topA Y319F* overexpression

Induced and non-induced *E. coli* DY330 *topA-SPA* pCA24 topA Y319F cultures were cultivated as described for ChIP-Seq (see ***EcTopoI ChIP-Seq***). RNA was extracted from 2 mL of culture using ExtractRNA reagent (Evrogen). RNA sample (90 µg) was treated with 5 U of DNase I (ThermoFisher Scientific) for 30 min at 37ºC and RNA was purified with RNAClean XP beads (Beckman Coulter). cDNA was synthesized from 7 µg of RNA using Maxima Reverse Transcriptase with random hexamer primers (Thermo Fisher Scientific). qPCR was performed using Maxima SYBR green qPCR master mix (Thermo Fisher Scientific) on CFX96 Real-Time System (Bio-Rad). For primers, see **Supplementary Table 1**. Expression levels of the studied genes (*gyrA*, *gyrB*, *parC*, *parE*, *topB*, *rnhA*, *rnhB*) were normalized relative to the transcription level of *rho*.

Probing the RpoC enrichment in response to CTD overexpression

Induced and non-induced *E. coli* DY330 *rpoC-TAP* pCA24 14kDa CTD cultures were prepared as described for ChIP-Seq (see ***EcTopoI ChIP-Seq***) and ChIP was performed as described for RpoC ChIP-Seq. qPCR was performed using Maxima SYBR green qPCR master mix (Thermo Fisher Scientific) on CFX96 Real-Time System (Bio-Rad). For primers, see **Supplementary Table 1**.

Supplementary Tables

Supplementary Table 1. List of oligonucleotides used for cloning, recombineering, qPCR, RT-qPCR, oligonucleotides used in binding and cleavage *in vitro* experiments

Primer	Sequence	The length of amplicon, bp	Description
topA_NcoI_fw	GTAAGGTGACCATGGGTAAAGCTCTTG	2508	*topA* amplification fragment 1
NcoI_mut_rev	GGCTAAAGCGAACCACGGTCTTATTACC		
NcoI_mut_fw	GGTAATAAGACCGTGGTTCGCTTTAGCC	170	*topA* amplification fragment 2. StrepII-coding sequence indicated by capitals.
topA_HindIII_strepII_rev	atataaagcttttaTTTTTCGAACTGCGGGTGGCTCCAcgcgcttttttttccttcaacccatttgccatc		
topA_NdeI_fw	GTAAGGTGCatATGGGTAAAGCTCTTGTCATCG	2654	*topA* amplification
topA_strepII_HindIII_rev	atatcaagcttTTATTTTTCGAACTGCGGGTGGCTCCAC		
TopoA_14_kDa_CTD_BamHI_fw	GGATCGCATCACCATCACCATCACGGATCCGGCGAAGTGGCACCACCG	423	EcTopoI CTD amplification
TopoA_CTD_HindIII_rev	aacaggagtccaagctcagctaattaagctTTATTTTTTTCCTTCAACCCATTTGCC		
pCA24_core_NcoI_rev	AATCTAACCATGGATCCGTGATGGTGATGGTGATGC	4515	Amplification of the pCA24 core
pCA24_core_HindIII_fw	AAACTGGGGCACAGATGAAGCTTAATTAGC		
topA_Y319F_fw	TATATCACTTTCATGCGTACCGACTCCACTAACC	7139	Introduction of Y319F mutation into pCA24 topA_strepII plasmid.
topA_Y319F_for	AGTCGGTACGCATGAAAGTGATATAGCCTG		
topA_XbaI_RBS_fw	TatataactctagaaataattttgtttaactttaagaaggagatatacCatgggcagcagccatcatcatcatcatcacagcagcggcctggtgccgcgcggcagccataTGGGTAAAGCTCTTGTCATCG	470	Introduction of G116S and M320V mutations in *topA*. Introduction of C-terminal strepII-tag. Cloning into pBAD33.
topA_G116S_out_rev	ATGCAATGGCTTCAGATTCGCGGTCAAGGTCGG		
topA_G116S_in_for	TTGACCGCGAATCTGAAGCCATTGCATGGCACC	636	
topA_M320V_in_rev	AGTCGGTACGCACGTAAGTGATATAGCCTGCTTCATAC		
topA_M320V_out_for	ATCACTTACGTGCGTACCGACTCCACTAACC	1691	
topA_strepII_HindIII_rev	atatcaagcttTTATTTTTCGAACTGCGGGTGGCTCCAC		
dps_850_F	CGGTGTAGAGGAAAAGTAGCGAGAAATTCTGC	124	Sequence under the

dps_850_R	ATGGGGTCTACGCTGACAGTACG		highest EcTopoI ChIP-Seq peak. Used for ChIP-qPCR and EMSA: *dps* region amplicon.
potF_895_F	CGGGAGAAAGTTCTCTTTTCTTACACCG	125	Sequence under one the highest EcTopoI ChIP-Seq peak. Used for ChIP-qPCR and EMSA: *potF* region amplicon.
potF_895_R	GCGGTCATATCTCTTTCCTTCTGAAAGTTCG		
L_2394_F	CCAGTACCAACAGTGCAGAGATC	110	Region with low signal of EcTopoI ChIP-Seq. Used for ChIP-qPCR and EMSA: *nuoN* region amplicon.
L_2395_R	GGTGAGCCTGTATCTTCACGC		
topA_delta_topA66_kanR_F	CACCACCGAAAGAAGATCCGGTGCCATTAC CTGAGC TGCCGTGCGAAAAATAATAGTGATCGTGTA GGCTG GAGCTGCTTC	1613	For amplification of resistance cassette for genome editing by recombineering. topA_SPA_kanR_cysB_R is universal for all the three forward primers.
topA_delta_14kDa_kanR_F	GTAAATACATGGCCTGCACCAACGAAGAGT GTAAAA ACACACGTAAGATTTTACGTAACTAATAGT GAATCGT GTAGGCTGGAGCTGCTTC	1623	
topA_delta_30kDa_kanR_F	AGTTAGATAAAGCTGAAAAAGATCCGGAA G AGGGTGGTATGCGCCCGAACTAATAGTGAA TCGTGTAGGCTGGAGCTGCTTC	1614	
topA_SPA_kanR_cysB_R	ATGCAATAAAAAAGGGCCGCTTTCGCGACC CTTTGTTTATAAAAACCTGACAGAATTAAA G GTGAAGAAGTAAAACATATGA ATATCCTCC		
topA_8_F1	GTTGAGTCCCCGGCAAAAGCC	138	To assess transcripts density over long transcription units with RT-qPCR and RNAP enrichment
topA_53_R1	GGTGGAGGTAGAGTCGGCACTC		
topA_73_F2	GGTTGACCCGTGGCACAATTGG	138	
topA_118_R2	GCTTCCCCTTCGCGGTCAAGG		
topA_146_F3	CGATCCGCCAGGCATTTAACAAACC	137	
topA_191_R3	CAGGCCACGAGCGATCTTTTTCC		
topA_303_F4	GATGGCGCAGCGTTTGTATGAAGC	139	
topA_352_R4	CGGACTTTCCGGCAGATATTTCTTACC		

topA_476_F5	CCGTTTCAGTGAAGCATCGCTGG	145	with ChIP-qPCR upon uncoupling of EcTopoI:RNAP complex by overexpression of EcTopoI 14kDa CTD: *topA* (~2600 bp).
topA_523_R5	GCCCATTTTTCCGCATAGAAACGACG		
topA_589_F6	GATGGTTCTGACCAGCATTGACTGC	138	
topA_633_R6	ACCAGGTTAATGGTGGTTTTGCAACG		
topA_694_F7	GGGCGAATTCCGCATTAAAGGTTATGAC	131	
topA_740_R7	CGTGTGTTTTTACACTCTTCGTTGGTGC		
Operon_1_rpmH_4_F1 (rpmH_1_F)	GCACTTTTCAACCGTCTGTACTGAAGCG	140	To assess transcripts density over long transcription units with RT-qPCR and RNAP enrichment with ChIP-qPCR upon uncoupling of EcTopoI:RNAP complex by overexpression of EcTopoI 14kDa CTD: *rpmH-rnpA-yidD-yidC* (~2200 bp).
Operon_1_rpmH_47_R1 (rpmH_1_R)	GCTTTATTACTTAGAAACGGTCAGACGAGCG		
Operon_1_rnpA_44_F2 (rpmH_2_F)	CATCCCCGTATCGGTCTTACAGTCG	138	
Operon_1_rnpA_R89 (rpmH_2_R)	CGCCACCACCACGAAATCCATAGC		
Operon_1_yidD_37_F3 (rpmH_3_F)	GGAGTGATAAAAGGCAGTTGGTTGACG	141	
Operon_1_yidC_9_R3 (rpmH_3_R)	CTAAAAGATTGCGTTGCGAATCCATCG		
Operon_1_yidC_67_F4 (rpmH_4_F)	CGTGCTTGATCTGACCATCAACACC	140	
Operon_1_yidC_113_R4 (rpmH_4_R)	CGCTCTGTGCCTGATAAATAAACTGCG		
Operon_1_yidC_166_F5 (rpmH_5_F)	GTCCTGAAACGTGGTGATTACGCTGTC	128	
Operon_1_yidC_208_R5 (rpmH_5_R)	GTATCGAGATGCGGTGGCAGAGTG		
Operon_1_yidC_238_F6 (rpmH_6_F)	TGCCGATAACGAAAACCTGAACATCTCTTC	139	
Operon_1_yidC_284_R6 (rpmH_6_R)	GGCGATGCCGTTACCCAGATTAGC		
Operon_1_yidC_362_F7 (rpmH_7_F)	ACCTTTATCGTTCGTGGCATCATGTACC	135	
Operon_1_yidC_401_R7 (rpmH_7_R)	CTGGCTGATACGCTGTTTGTCATCG		
Operon_1_yidC_482_F8 (rpmH_8_F)	TCGCCGACCACAGTGACCGACC	139	
Operon_1_yidC_528_R8 (rpmH_8_R)	GCTGAATAATGGTTACCAGGTTGCTGACG		
BW25113_topA_wt_F	GCCGCTTTCGCGACCCTTTG	103	To assess the composition of a mixed culture undergo the competition test (*E. coli* BW25113 vs BW25113 *topA* mutant) by qPCR.
BW25113_topA_wt_R	CGACTGGCTGGTCAGCATTTTATGTTGATGG		
BW25113_topA_delta11_SPA_F	GAGCTGCCGTGCGAAAAATCCATG	106	
BW25113_SPA_qPCR_R	CAAGTGCCCCGGAGGATGAGATTTTC		
BW25113_topA_delta14_SPA_F	ACACACGTAAGATTCTGCGCAATTCCATG	111	

BW25113_SPA_qPCR_R	CAAGTGCCCCGGAGGATGAGATTTC		
EcTopoI_Consensus_F_Cy5	Cy5GCGTTTTTTTCTTCATTATTTTTGCG		Oligonucleotides used in binding experiments with EcTopoI. Oligos carrying Cy5 labels on 5'-end.
EcTopoI_Consensus_R_Cy5	Cy5CGCAAAAATAATGAAGAAAAAAACGC		
Random_F_Cy5	Cy5GCGCACGTAATCCGCAGTGCGGTGCG		
Random_R_Cy5	Cy5CGCTCCGCACTGCGGATTACGTGCGC		
Poly-T_Cy5	Cy5GCGTTTTTTTTTTTTTTTTTTTTTGCG		
Poly-A_Cy5	Cy5CGCAAAAAAAAAAAAAAAAAAAAACGC		
EcTopoI_Consensus_F	GCGTTTTTTTCTTCATTATTTTTGCG		Oligonucleotides used in binding experiments with EcTopoI. Oligos are label-free.
EcTopoI_Consensus_R	CGCAAAAATAATGAAGAAAAAAACGC		
Random_F	GCGCACGTAATCCGCAGTGCGGTGCG		
Random_R	CGCTCCGCACTGCGGATTACGTGCGC		
Poly-T	GCGTTTTTTTTTTTTTTTTTTTTTGCG		
Poly-A	CGCAAAAAAAAAAAAAAAAAAAAACGC		
rnhA_F	GGGTTACGGCGCTATTTTACGCTATCG	135	*rnhA* RT-qPCR
rnhA_R	ACTTCGCAATGTTCTTTTAACGCCTCC		
rnhB_F	AGCTGGTTGCGGGTGTGGATG	132	*rnhB* RT-qPCR
rnhB_R	GGCGTTTTTCGCTCAGCTTTTTGG		
gyrA_F	GGTATCGCCGTAGGTATGGCAACC	123	*gyrA* RT-qPCR
gyrA_R	CGGGATGTGTTCCATCAGCCCTTC		
gyrB_F	GCACCACATGGTATTCGAGGTGGTAG	146	*gyrB* RT-qPCR
gyrB_R	CCGGGTGAATACCGGTCGGAATG		
parC_F	GGACGTGGTTCAGTGCGTATGC	148	*parC* RT-qPCR
parC_R	CGTCAACCATCGGCAGCTTTTTGTTG		
parE_F	GGTGTACCGGCGGTTGAACTG	142	*parE* RT-qPCR
parE_R	CGTTAACTTCTACGCGCTTCGACAGG		
topB_F	GGGCAATTGCTGGTGGATGAAGTG	139	*topB* RT-qPCR
topB_R	CACTGTTGGAACGAAGACGGTCG		
rho_F	GTTCCGGCGTCAGGTAAAGTGTTG	123	*rho* RT-qPCR
rho_R	CGTCGCGATAATGGTCAGGCTG		

Supplementary Table 2. List of NGS datasets used in the current study

Description	Study
Total RNA-Seq for exponentially growing *E. coli* DY330 culture in LB. Performed in triplicate. GSM5509255-GSM5509257	Current study
E. coli RNAP (RpoC) ChIP-Seq performed with exponentially growing *E. coli* DY330 *rpoC-TAP* culture in LB. Single replicate. GSM5538289, GSM5538290	Current study
E. coli TopoI ChIP-Seq performed with exponentially growing *E. coli* DY330 *topA-SPA* culture in LB. Performed in triplicate. GSM5514277-GSM5514282	Current study
E. coli TopoI ChIP-Seq performed with exponentially growing *E. coli* DY330 *topA-SPA* culture in LB. Culture was pre-treated with 100 mkg/ml rifampicin for 20 min before crosslinking. Performed in triplicate. GSM5514289-GSM5514294	Current study
E. coli TopoI ChIP-Seq performed with exponentially growing *E. coli* DY330 *topA-SPA* culture in LB. CTD expression was induced from pCA24 14kDa CTD when OD_{600} reached 0.2. Performed in triplicate. GSM5514283-GSM5514288	Current study
E. coli TopoI ChIP-Seq performed with exponentially growing *E. coli* DY330 *topA-SPA* culture in LB. CTD expression was induced from pCA24 14kDa CTD when OD_{600} reached 0.2. Culture was pre-treated with 100 mkg/ml rifampicin for 20 min before crosslinking. Performed in duplicate. GSM5514295-GSM5514298	Current study
E. coli TopoI Topo-Seq for cleavage sites mapping performed with exponentially growing *E. coli* DY330 *topA-SPA* culture in LB. Performed in triplicate. GSM5529907-GSM5529918	Current study
DRIP-Seq for R-loops mapping performed with exponentially growing *E. coli* DY330 *topA-SPA* culture in LB. Performed in triplicate. GSM5514654-GSM5514659	Current study
DRIP-Seq for R-loops mapping performed with exponentially growing *E. coli* DY330 *topA-SPA* culture in LB. Culture was pre-treated with 100 mkg/ml rifampicin for 20 min before cells harvesting. Performed in triplicate. GSM5514666-GSM5514671	Current study
DRIP-Seq for R-loops mapping performed with exponentially growing *E. coli* DY330 *topA-SPA* culture in LB. CTD expression was induced from pCA24 14kDa CTD when OD_{600} reached 0.2. Performed in triplicate. GSM5514660-GSM5514665	Current study

DRIP-Seq for R-loops mapping performed with exponentially growing *E. coli* DY330 *topA-SPA* culture in LB. CTD expression was induced from pCA24 14kDa CTD when OD_{600} reached 0.2. Culture was pre-treated with 100 mkg/ml rifampicin for 20 min before cells harvesting. Performed in triplicate. GSM5514672-GSM5514676	Current study
WGS of *E. coli* strains with truncated versions of *topA* gene constructed by lambda-red recombineering. PRJNA757761	Current study
E. coli DNA gyrase Topo-Seq performed with exponentially growing *E. coli* DY330 *gyrA-SPA MuSGS* culture in LB. Ciprofloxacin was used to trap covalent intermediate complexes between gyrase and DNA. Performed in triplicate. GSM3273139-GSM3273144	Sutormin D, Rubanova N, Logacheva M, Ghilarov D et al. Single-nucleotide-resolution mapping of DNA gyrase cleavage sites across the Escherichia coli genome. Nucleic Acids Res 2019 Feb 20;47(3):1373-1388. PMID: 30517674
E. coli DNA gyrase Topo-Seq performed with exponentially growing *E. coli* DY330 *gyrA-SPA MuSGS* culture in LB. Culture was pre-treated with 100 mkg/ml rifampicin for 20 min before adding the ciprofloxacin to trap covalent intermediate complexes between gyrase and DNA. Performed in triplicate. GSM3273145-GSM3273150	Sutormin D, Rubanova N, Logacheva M, Ghilarov D et al. Single-nucleotide-resolution mapping of DNA gyrase cleavage sites across the Escherichia coli genome. Nucleic Acids Res 2019 Feb 20;47(3):1373-1388. PMID: 30517674
E. coli RNAP (RpoB) ChIP-Seq performed with exponentially growing *E. coli* MG1655 culture in LB. Single replicate. GSM613808	Kahramanoglou C, Seshasayee AS, Prieto AI, Ibberson D et al. Direct and indirect effects of H-NS and Fis on global gene expression control in Escherichia coli. Nucleic Acids Res 2011 Mar;39(6):2073-91. PMID: 21097887
E. coli MG1655 RNAP (RpoC) ChIP-chip performed with exponentially growing *E. coli* MG1655 culture in M9 medium. The culture was pre-treated with Rif before the experiment. Single replicate. GSM351003	Mooney RA, Davis SE, Peters JM, Rowland JL et al. Regulator trafficking on bacterial transcription units in vivo. Mol Cell 2009 Jan 16;33(1):97-108.
C. crescentus GapR ChIP-Seq (GapR-Seq) for positive supercoiling mapping performed with exponentially growing *E. coli* MG1655 culture in LB. Performed in duplicate. GSM4628311-GSM4628314	Monica S Guo, Ryo Kawamura, Megan L Littlehale, John F Marko, Michael T Laub. High-resolution, genome-wide mapping of positive supercoiling in chromosomes. eLife 2021;10:e67236. PMID: 34279217
Psora-Seq for negative supercoiling mapping performed with exponentially growing *E. coli* MG1655 culture in LB. Six replicates. SRR17974444-SRR17974449, SRR17974460, SRR17974471, SRR17974480-SRR17974483	Bryan J Visser, Sonum Sharma, Po J Chen, Anna B McMullin, Maia L Bates, David Bates. Psoralen mapping reveals a bacterial genome supercoiling landscape dominated by transcription. NAR 2022; https://doi.org/10.1093/nar/gkac244. PMID: 35420137
Mycobacterium tuberculosis DNA-gyrase (GyrA) ChIP-Seq performed with exponentially growing *M. tuberculosis* Ra culture. Single replicate. GSM2538162	Ahmed W, Sala C, Hegde SR, Jha RK et al. Transcription facilitated genome-wide recruitment of topoisomerase I and DNA gyrase. PLoS Genet 2017 May;13(5):e1006754. PMID: 28463980

Mycobacterium tuberculosis RNAP (RpoB) ChIP-Seq performed with exponentially growing *M. tuberculosis* H37Rv culture. Performed in duplicate. GSM1003214, GSM1003215	Uplekar S, Rougemont J, Cole ST, Sala C. High-resolution transcriptome and genome-wide dynamics of RNA polymerase and NusA in Mycobacterium tuberculosis. Nucleic Acids Res 2013 Jan;41(2):961-77. PMID: 23222129
Total RNA-Seq performed for exponentially growing *M. tuberculosis* H37Rv culture. Performed in duplicate. GSM1003224, GSM1003225	Uplekar S, Rougemont J, Cole ST, Sala C. High-resolution transcriptome and genome-wide dynamics of RNA polymerase and NusA in Mycobacterium tuberculosis. Nucleic Acids Res 2013 Jan;41(2):961-77. PMID: 23222129
Mycobacterium smegmatis RNAP (RpoB) ChIP-Seq performed with exponentially growing *M. smegmatis* Mc2155 culture. Performed in duplicate. GSM1171544, GSM1171545	Landick R, Krek A, Glickman MS, Socci ND et al. Genome-Wide Mapping of the Distribution of CarD, RNAP σA, and RNAP β on the Mycobacterium smegmatis Chromosome using Chromatin Immunoprecipitation Sequencing. Genom Data 2014 Dec;2:110-113. PMID: 25089258
Mycobacterium smegmatis TopoI ChIP-Seq performed with exponentially growing *M. smegmatis* Mc2155 culture. Single replicate. SRX4970107	Rani P et al., "Genome-wide mapping of Topoisomerase I activity sites reveal its role in chromosome segregation.", Nucleic Acids Res, 2019 Feb 20;47(3):1416-1427
Mycobacterium smegmatis ChIP-Seq mock data for *M. smegmatis* Mc2155 culture. Performed in duplicate. GSM4274349, GSM4274350	Feng S, Liu Y, Liang W, El-Sayed Ahmed MAE et al. Involvement of Transcription Elongation Factor GreA in Mycobacterium Viability, Antibiotic Susceptibility, and Intracellular Fitness. Front Microbiol 2020;11:413.
Total RNA-Seq performed for exponentially growing *M. smegmatis* Mc2155 culture. Performed in duplicate. GSM2756262, GSM2756263	Li X, Mei H, Chen F, Tang Q et al. Transcriptome Landscape of Mycobacterium smegmatis. Front Microbiol 2017;8:2505. PMID: 29326668
Streptococcus pneumoniae TopoI and RNAP ChIP-Seqs performed for exponentially growing culture. Single replicates. SRR12427932, SRR12427936, SRR12427942	Maria-Jose Ferrandiz, Pablo Hernandez, Adela G. de la Campa, Genome-wide proximity between RNA polymerase and DNA topoisomerase I supports transcription in Streptococcus pneumoniae. PLOS Genetics 2021. PMID: 33930020

Supplementary Table 3. Kd values identified for the binding of oligonucleotides to EcTopoI *in vitro* using EMSA and MST methods

**Oligonucleotide**	Kd Replicate 1 (EMSA), nM	Kd Replicate 2 (EMSA), nM	Kd Replicate 3 (EMSA), nM	**Kd mean (EMSA), nM**	**Kd std (EMSA), nM**	**Kd mean (MST), nM**	**Kd std (MST), nM**
Consensus F	290	140	140	190	87	21	7
Consensus R	290	190	140	207	76	27	1
Random F	390	290	390	357	58	321	11
Random R	390	590	390	457	115	328	38
Poly-T	190	390	290	290	100	62	22
Poly-A	290	190	190	223	58	40	2

Supplementary References

1. Kahramanoglou, C. *et al.* Direct and indirect effects of H-NS and Fis on global gene expression control in Escherichia coli. *Nucleic Acids Res.* **39**, 2073–2091 (2011).
2. Santos-Zavaleta, A. *et al.* A unified resource for transcriptional regulation in Escherichia coli K-12 incorporating high-throughput-generated binding data into RegulonDB version 10.0. *BMC Biol.* **16**, 1–12 (2018).
3. Peabody, M. A., Laird, M. R., Vlasschaert, C., Lo, R. & Brinkman, F. S. L. PSORTdb: Expanding the bacteria and archaea protein subcellular localization database to better reflect diversity in cell envelope structures. *Nucleic Acids Res.* **44**, D663–D668 (2016).
4. Sutormin, D., Rubanova, N., Logacheva, M., Ghilarov, D. & Severinov, K. Single-nucleotide-resolution mapping of DNA gyrase cleavage sites across the Escherichia coli genome. *Nucleic Acids Res.* **47**, 1–16 (2019).
5. Allali, N., Afif, H., Couturier, M. & Van Melderen, L. The highly conserved TldD and TldE proteins of Escherichia coli are involved in microcin B17 processing and in CcdA degradation. *J. Bacteriol.* **184**, 3224–3231 (2002).
6. Kulakovskiy, I. V., Boeva, V. A., Favorov, A. V. & Makeev, V. J. Deep and wide digging for binding motifs in ChIP-Seq data. *Bioinformatics* **26**, 2622–2623 (2010).
7. Zhang, Y. *et al.* Model-based analysis of ChIP-Seq (MACS). *Genome Biol.* **9**, R137.1-R137.9 (2008).
8. Gupta, S., Stamatoyannopoulos, J. A., Bailey, T. L. & Noble, W. S. Quantifying similarity between motifs. *Genome Biol.* **8**, (2007).
9. Li, X. *et al.* Transcriptome landscape of Mycobacterium smegmatis. *Front. Microbiol.* **8**, 1–16 (2017).
10. Landick, R., Krek, A., Glickman, M. S., Socci, N. D. & Stallings, C. L. Genome-wide mapping of the distribution of CarD, RNAP σA, and RNAP β on the Mycobacterium smegmatis chromosome using chromatin immunoprecipitation sequencing. *Genomics Data* **2**, 110–113 (2014).
11. Rani, P. & Nagaraja, V. Genome-wide mapping of Topoisomerase I activity sites reveal its role in chromosome segregation. *Nucleic Acids Res.* **47**, 1416–1427 (2018).
12. Uplekar, S., Rougemont, J., Cole, S. T. & Sala, C. High-resolution transcriptome and genome-wide dynamics of RNA polymerase and NusA in Mycobacterium tuberculosis. *Nucleic Acids Res.* **41**, 961–977 (2013).
13. Ahmed, W. *et al.* Transcription facilitated genome-wide recruitment of topoisomerase I and DNA gyrase. *PLoS Genet.* **13**, e1006754 (2017).
14. Ferrandiz, M.-J., Hernandez, P. & de la Campa, A. G. Genome-wide proximity between RNA polymerase and DNA topoisomerase I supports transcription in Streptococcus pneumoniae. *PLoS Genet.* **4**, 1–21 (2021).
15. Butland, G. *et al.* Interaction network containing conserved and essential protein complexes in Escherichia coli. *Nature* **433**, 531–37 (2005).
16. Langmead, B., Trapnell, C., Pop, M. & Salzberg, S. L. Ultrafast and memory-efficient alignment of short DNA sequences to the human genome. *Genome Biol.* **10**, R25.1-R25.10 (2009).
17. Ramírez, F., Dündar, F., Diehl, S., Grüning, B. A. & Manke, T. DeepTools: A flexible

platform for exploring deep-sequencing data. *Nucleic Acids Res.* **42**, 187–191 (2014).

18. Zhang, Y. *et al.* Model-based Analysis of ChIP-Seq (MACS). *Genome Biol.* **9**, R137.1-R137.9 (2008).

19. Thorvaldsdóttir, H., Robinson, J. T. & Mesirov, J. P. Integrative Genomics Viewer (IGV): High-performance genomics data visualization and exploration. *Brief. Bioinform.* **14**, 178–192 (2013).

20. Li, H. & Durbin, R. Fast and accurate long-read alignment with Burrows-Wheeler transform. *Bioinformatics* **26**, 589–595 (2010).

21. Li, H. *et al.* The Sequence Alignment/Map format and SAMtools. *Bioinformatics* **25**, 2078–2079 (2009).

22. Karp, P. D. *et al.* The BioCyc collection of microbial genomes and metabolic pathways. *Brief. Bioinform.* **20**, 1085–1093 (2018).

23. Wang, L., Wang, S. & Li, W. RSeQC: Quality control of RNA-seq experiments. *Bioinformatics* **28**, 2184–2185 (2012).

24. Feng, S. *et al.* Involvement of Transcription Elongation Factor GreA in Mycobacterium Viability, Antibiotic Susceptibility, and Intracellular Fitness. *Front. Microbiol.* **11**, 1–15 (2020).

25. Guo, M. S., Kawamura, R., Littlehale, M., Marko, J. F. & Laub, M. T. High-resolution, genome-wide mapping of positive supercoiling in chromosomes. *Elife* **10**, (2021).

26. Mooney, R. A. *et al.* Regulator trafficking on bacterial transcription units in vivo. *Mol. Cell* **33**, 97–108 (2009).
