# Supplementary material for: Interaction between transcribing RNA polymerase and topoisomerase I prevents R-loop formation in E. coli
Source: Nat Commun. 2022 Aug 4;13:4524. doi: 10.1038/s41467-022-32106-5 (PMC9352719; doi:10.1038/s41467-022-32106-5)
Supplement: Supplementary file 3 — Reporting Summary [file 41467_2022_32106_MOESM3_ESM.pdf]

nature portfolio

Corresponding author(s): Konstantin Severinov

Last updated by author(s): 29/06/22

Reporting Summary

Nature Portfolio wishes to improve the reproducibility of the work that we publish. This form provides structure for consistency and transparency in reporting. For further information on Nature Portfolio policies, see our Editorial Policies and the Editorial Policy Checklist.

Statistics

For all statistical analyses, confirm that the following items are present in the figure legend, table legend, main text, or Methods section.

n/a	Confirmed	
☐	☒	The exact sample size (*n*) for each experimental group/condition, given as a discrete number and unit of measurement
☒	☐	A statement on whether measurements were taken from distinct samples or whether the same sample was measured repeatedly
☐	☒	The statistical test(s) used AND whether they are one- or two-sided<br>*Only common tests should be described solely by name; describe more complex techniques in the Methods section.*
☒	☐	A description of all covariates tested
☒	☐	A description of any assumptions or corrections, such as tests of normality and adjustment for multiple comparisons
☐	☒	A full description of the statistical parameters including central tendency (e.g. means) or other basic estimates (e.g. regression coefficient) AND variation (e.g. standard deviation) or associated estimates of uncertainty (e.g. confidence intervals)
☐	☒	For null hypothesis testing, the test statistic (e.g. *F*, *t*, *r*) with confidence intervals, effect sizes, degrees of freedom and *P* value noted<br>*Give P values as exact values whenever suitable.*
☒	☐	For Bayesian analysis, information on the choice of priors and Markov chain Monte Carlo settings
☒	☐	For hierarchical and complex designs, identification of the appropriate level for tests and full reporting of outcomes
☐	☒	Estimates of effect sizes (e.g. Cohen's *d*, Pearson's *r*), indicating how they were calculated

Our web collection on statistics for biologists contains articles on many of the points above.

Software and code

Policy information about availability of computer code

Data collection

Initial processing of sequencing data (base-calling) was performed with Illumina software HCS v3.3.76 pre-installed in Illumina HiSeq 4000 with standard parameters. Microscale thermophoresis data was collected with Nanotemper Monolith controlled by MO.Control v2. Microscopy image acquisition was controlled by NIS-Elements BR 4.51.01.

Data analysis

Sequencing reads were filtered and trimmed with Trimmomatic v0.38 with the following parameters: ILLUMINACLIP:Adapters:2:30:10 LEADING:0 TRAILING:0 SLIDINGWINDOW:4:0 MINLEN:30.
Sequencing reads were aligned using bwa mem v0.7.17-r1188 (default parameters). For RNAP ChIP-Seq read were aligned using bowtie v1.2.2.
For RNAP ChIP-Seq quality assessment was calculated using DeepTools v2.5.0. Log2 ratio profiles of IP to input samples were calculated using BAMcompare tool from DeepTools.
SAM, BAM files were prepared with Samtools v1.10 (default parameters). BED files were prepared with Samtools depth v1.10 with -d 0 option.
Sequencing data was visualized with IGV v2.7.2.
SNPs and short indels were called with bcftools (v1.10.2) mpileup (default parameters) and call (-c option).
For EcTopoI ChIP-Seq data, peak calling was performed with MACS2 v 2.2.6 with following parameters: nomodel, Q-value<0.001.
For RNAP ChIP-Seq, regions with fold enrichment > 3 were defined as peaks.
Motif identification was performed by ChIPMunk V8 (default parameters).
For RNA-Seq data analysis FPKM_count.py program from the RSeQC package (v2.6.4) was used for FPKM and genes expression level calculations with following parameters: -q 25.
Coverage depth and enrichment tracks were further analyzed using custom python scripts available from github repositories:
For TopoI and RNAP ChIP-Seq data analysis: https://github.com/sutormin94/TopoI_ChIP-Seq
For TopoI Topo-Seq data analysis: https://github.com/sutormin94/TopoI_Topo-Seq
For DRIP-Seq data analysis: https://github.com/sutormin94/E_coli_DRIP-Seq_analysis
For RNA-Seq data analysis: https://github.com/sutormin94/E_coli_RNA-Seq_analysis

Analysis of microscale thermophoresis data was performed with MO.Affinity Analysis v3. Microscopy images analysis (cell length quantification) was performed with ImageJ2 v2.35.

For manuscripts utilizing custom algorithms or software that are central to the research but not yet described in published literature, software must be made available to editors and reviewers. We strongly encourage code deposition in a community repository (e.g. GitHub). See the Nature Portfolio guidelines for submitting code & software for further information.

Data

Policy information about availability of data

All manuscripts must include a data availability statement. This statement should provide the following information, where applicable:

- Accession codes, unique identifiers, or web links for publicly available datasets
- A description of any restrictions on data availability
- For clinical datasets or third party data, please ensure that the statement adheres to our policy

Sequencing data for E. coli RNA-Seq (GSE181687 [https://www.ncbi.nlm.nih.gov/geo/query/acc.cgi?acc=GSE181687]), E. coli TopoI ChIP-Seq and Topo-Seq (GSE181915 [https://www.ncbi.nlm.nih.gov/geo/query/acc.cgi?acc=GSE181915] and GSE182473 [https://www.ncbi.nlm.nih.gov/geo/query/acc.cgi?acc=GSE182473], respectively), E. coli RpoC ChIP-Seq (GSE182850 [https://www.ncbi.nlm.nih.gov/geo/query/acc.cgi?acc=GSE182850]), E. coli DRIP-Seq (GSE181945 [https://www.ncbi.nlm.nih.gov/geo/query/acc.cgi?acc=GSE181945]) were deposited in GEO with corresponding dataset accession numbers. Sequencing data for E. coli topA mutants' whole-genome sequencing was deposited in SRA (PRJNA757761 [https://www.ncbi.nlm.nih.gov/bioproject/757761]). See full list of NGS datasets used in the study in Supplementary Table 2. E. coli W3110 genome annotations with ORFs, operons, and TUs were obtained from Ensembl Bacteria (Howe et al., 2020), DOOR (Mao et al., 2009), and EcoCyc (Karp et al., 2018) databases, respectively. Information about the subcellular localization of E. coli proteins was retrieved from PSORTdb 4.0 (Peabody et al., 2016). Annotations of promoters and transcription factor sites were obtained from RegulonDB (Santos-Zavaleta et al., 2019). Source data are provided with this paper. Source data are provided with this paper.

Field-specific reporting

Please select the one below that is the best fit for your research. If you are not sure, read the appropriate sections before making your selection.

[X] Life sciences   [ ] Behavioural & social sciences   [ ] Ecological, evolutionary & environmental sciences

For a reference copy of the document with all sections, see nature.com/documents/nr-reporting-summary-flat.pdf

Life sciences study design

All studies must disclose on these points even when the disclosure is negative.

Sample size	No sample size calculations were performed. NGS-based experiments (ChIP-Seq, Topo-Seq, DRIP-Seq, RNA-Seq) were performed in triplicates where possible according to the common design used in the field.
Data exclusions	For qPCR data, a technical replicate was excluded if Ct differs by more than 1 cycle from the other two replicates. No data exclusion was performed in other experiments.
Replication	ChIP-Seq, Topo-Seq, RNA-Seq, DRIP-Seq experiments were performed in triplicates, all attempts were successful. Growth curves, CFU-counting, plasmid topology experiments, SOS-response detection experiments were performed in triplicates, all attempts were successful. RNAP pull-down experiments were repeated five times, all replicates were successful.
Randomization	Was not performed intentionally, because all experiments were performed with large populations comprised of non-labeled cells.
Blinding	Was not performed, as automatic ChIP-Seq, Topo-Seq, DRIP-Seq, RNA-Seq data analyses give unbiased measurements.

Reporting for specific materials, systems and methods

We require information from authors about some types of materials, experimental systems and methods used in many studies. Here, indicate whether each material, system or method listed is relevant to your study. If you are not sure if a list item applies to your research, read the appropriate section before selecting a response.

Materials & experimental systems

n/a	Involved in the study	
[ ]	[X]	Antibodies
[X]	[ ]	Eukaryotic cell lines
[X]	[ ]	Palaeontology and archaeology
[X]	[ ]	Animals and other organisms
[X]	[ ]	Human research participants
[X]	[ ]	Clinical data
[X]	[ ]	Dual use research of concern

Methods

n/a	Involved in the study	
[ ]	[X]	ChIP-seq
[X]	[ ]	Flow cytometry
[X]	[ ]	MRI-based neuroimaging

Antibodies

Antibodies used

S9.6 antibodies (ENH001, Kerafast) for R-loops dot-blot and DRIP-Seq experiments. For dot-blot, antibodies were diluted 1:1850.
Anti-FLAG M2 antibodies conjugated with agarose gel (A2220, Sigma Aldrich). Was used as an affinity gel, dilution is not applicable.
Anti-FLAG antibodies produced in rabbits (Sigma Aldrich, F7425) in a 1:10000 dilution.
Secondary anti-rabbit antibodies produced in goat conjugated with HRP (Sigma Aldrich, A0545) in a 1:25000 dilution.
Secondary anti-mouse antibodies conjugated with HRP (Sigma Aldrich, A9044) in a 1:80000 dilution.

Validation

S9.6 are well-validated and widely used antibodies against R-loops: see Reference section in the manufacturer's site https://www.kerafast.com/productgroup/432/anti-dna-rna-hybrid-s96-antibody?ProductID=2082&gclid=Cj0KCQiA5OuNBhCRARIsACgaiqV3aWtVAZlrSn5_IbBvQzC_GzLdGzUUjrHjjZSAALPuRJELXwe2SCAaAsckEALw_wcB
Anti-FLAG M2 antibodies conjugated with agarose gel is a widely-used affinity gel: see Peer Reviewed papers section in the vendor's site: https://www.sigmaaldrich.com/RU/en/product/sigma/a2220
Anti-FLAG rabbit antibodies (Sigma Aldrich, F7425) are widely used: see Peer Reviewed papers section in the vendor's site: https://www.sigmaaldrich.com/RU/en/product/sigma/f7425

ChIP-seq

Data deposition

☒ Confirm that both raw and final processed data have been deposited in a public database such as GEO.

☒ Confirm that you have deposited or provided access to graph files (e.g. BED files) for the called peaks.

Data access links
May remain private before publication.

Sequencing data for:
E. coli TopoI ChIP-Seq (GSE181915): snmjiocadjextiv
E. coli RpoC ChIP-Seq (GSE182850): ongdqcywpxmjbwj
were deposited in GEO with corresponding dataset accession numbers and secure tokens to grant the access for Reviewers, respectively.

Files in database submission

Raw sequencing files (fastq.gz), coverage depth files (wig), fold enrichment files (wig), peak regions (NarrowPeak or BroadPeak), reference genome (fasta).

Genome browser session
(e.g. UCSC)

N/A

Methodology

Replicates

E. coli TopoI ChIP-Seq experiments were all performed in triplicates. One replicate was performed for E. coli RNAP ChIP-seq. As controls mock DNA (not enriched) was used.

Sequencing depth

ChIP-Seq data was sequenced in 150+150 bp paired-end mode. For IP and Mock samples on average 15,250,577 reads were obatined per sample (standard deviation 4,969,319 reads). On average not less than 95% of reads was uniquely mapped to the reference genome. For exact read numbers see below:

File name Total number of reads Number of uniquely mapped reads
EcTopoI_IP_1 14185998 14112208
EcTopoI_IP_2 7280866 6755200
EcTopoI_IP_3 12841744 12432998
EcTopoI_IP_CTD_1 12601352 12464687
EcTopoI_IP_CTD_2 14447942 14375148
EcTopoI_IP_CTD_3 12965370 12907795
EcTopoI_IP_CTD_Rif_1 13649570 13441980
EcTopoI_IP_CTD_Rif_2 11698840 11559453
EcTopoI_IP_Rif_1 11615028 11607030
EcTopoI_IP_Rif_2 13983124 13972265
EcTopoI_IP_Rif_3 11038776 10960453
Mock_1 20074666 19532634
Mock_2 15173466 8131953
Mock_3 8314628 7329280
Mock_CTD_1 21241856 20230702
Mock_CTD_2 23566670 22725722
Mock_CTD_3 19650456 19030435
Mock_CTD_Rif_1 16630998 16003404
Mock_CTD_Rif_2 23147160 22063079
Mock_Rif_1 20813026 19379255
Mock_Rif_2 19172682 18021242
Mock_Rif_3 23098264 21754250
RpoC_TAP_RNAP_wt_LB_IP 8814844 8805700
RpoC_TAP_RNAP_wt_LB_mock 10006520 10005668

Antibodies	Anti-FLAG M2 antibodies conjugated with agarose gel (A2220, Sigma Aldrich).
Peak calling parameters	Sequencing reads were aligned using bwa mem v0.7.17-r1188 (default parameters).<br>SAM, BAM files were prepared with Samtools v1.10 (default parameters). BED files were prepared with Samtools depth v1.10 with -d 0 option.<br>For EcTopoI ChIP-Seq data, peak calling was performed with MACS2 v 2.2.6 with following parameters: nomodel, Q-value<0.001.<br>For RNAP ChIP-Seq, regions with fold enrichment > 3 were defined as peaks.
Data quality	Peaks were called separately for all biological replicates. Final peaks shared between all 3 biological replicates (for TopoI ChIP-Seq experiments) were used for motif identification and other analyses.
Software	Motif identification was performed by ChIPMunk V8 (default parameters).<br>Coverage depth and enrichment tracks were further analyzed using custom python scripts stored in github repository: https://github.com/sutormin94/TopoI_ChIP-Seq
